# Polypharmacology‐Driven Discovery of ZAK‐I‐57: A Potent Multi‐Targeted Benzoxazinone Small Molecule for Hepatocellular Carcinoma Therapy

**DOI:** 10.1002/mco2.70291

**Published:** 2025-07-27

**Authors:** Shakeel Ahmad Khan, Huihai Yang, Fan Ying, Chin Ngok Chu, Terence Kin Wah Lee

**Affiliations:** ^1^ Department of Applied Biology and Chemical Technology The Hong Kong Polytechnic University Hung Hom Hong Kong SAR China; ^2^ State Key Laboratory of Chemical Biology and Drug Discovery The Hong Kong Polytechnic University Hong Kong SAR China

**Keywords:** benzoxazinone derivatives, hepatocellular carcinoma, multi‐targeted therapy, polypharmacology, tumor suppression

## Abstract

Hepatocellular carcinoma (HCC) is a deadly disease characterized by a high mortality rate and resistance to conventional therapies, highlighting the need for novel therapeutic interventions. Given the multifaceted nature of HCC pathogenesis, a multitargeted and polypharmacological approach is crucial for effective treatment. This study reports the potent multitargeted and polypharmacological properties of ZAK‐I‐57, a benzoxazinone derivative, as a potential therapeutic option for HCC. In cell‐based model, ZAK‐I‐57 demonstrated significant in vitro inhibition of proliferation in HCC cells. Utilizing PLC/PRF/5 tumor‐bearing and HCC patient‐derived tumor xenograft (PDTX) mouse models, we compared the efficacy of ZAK‐I‐57 with that of sorafenib, the current standard treatment. ZAK‐I‐57 demonstrated superior tumor suppressive effects at doses of 15 and 30 mg/kg, outperforming sorafenib. Western blot analysis revealed that ZAK‐I‐57 downregulated the oncogenic proteins EGFR and c‐Myc, while promoting apoptosis by increasing Bax and decreasing Bcl‐2 expression. Strikingly, ZAK‐I‐57 exhibited excellent ADMET properties, including high gastrointestinal absorption and good lipophilicity, along with an excellent safety profile, with no significant off‐target toxicity in vital organs. In summary, our findings highlight ZAK‐I‐57 as a new and promising multitarget therapeutic agent for HCC, warranting further clinical investigation to improve patient outcomes.

## 1 Introduction

Hepatocellular carcinoma (HCC) is the most prevalent primary liver cancer and among the leading causes of cancer‐related mortality worldwide. This malignancy is often associated with underlying chronic liver diseases, including hepatitis B and C infections, alcoholic liver disease, and nonalcoholic steatohepatitis [1, 2]. Despite advances in early detection and surgical interventions, the prognosis for HCC remains poor owing to its aggressive nature and high recurrence rates. Traditional chemotherapeutic approaches have limited efficacy, thus propelling the search for more effective treatment modalities [3, 4]. Molecular targeted therapy has revolutionized the therapeutic landscape for HCC by focusing on specific molecular targets that drive tumor growth and progression [5, 6]. These therapies aim to inhibit key signaling pathways implicated in HCC pathogenesis, such as the VEGF, PDGFR, and RAF/MEK/ERK pathways [7]. Agents such as sorafenib and lenvatinib have demonstrated clinical benefits by improving overall survival and delaying disease progression in patients with advanced HCC. However, the survival benefit of sorafenib is modest due to the development of resistance and surprisingly, only 20% of patients tolerate sorafenib, resulting in moderate‐to‐severe adverse effects, necessitating the exploration of novel therapeutic strategies [8, 9, 10]. Furthermore, the heterogeneity of HCC often renders single‐target therapies insufficient, as they fail to address the multifaceted nature of disease mechanisms [11].

To overcome these limitations, there is increasing interest in polypharmacology, which aims to concurrently modulate multiple molecular targets. Polypharmacological agents can bind to and functionally influence several proteins, providing a holistic approach to disease management. This strategy can be achieved through either combination therapy or the development of single compounds capable of multiple target interactions. Polypharmacology offers several advantages over traditional combination therapies, including superior pharmacokinetic and safety profiles, a lower likelihood of acquired resistance, and streamlined treatment regimens that enhance patient compliance [12, 13, 14]. The application of polypharmacology to molecular‐targeted therapies is particularly promising. For instance, polypharmacological compounds have shown efficacy in treating KRAS mutant non‐small cell lung cancers, which have proven refractory to conventional single‐target agents [12]. Despite these advancements, a significant challenge in polypharmacology remains the design of compounds that effectively inhibit multiple proteins with high potency [15]. Traditionally, the discovery of such agents has been serendipitous, often requiring substantial time and resources to identify suitable hit scaffolds [13]. However, recent progress in systems biology, system pharmacology, bioinformatics, machine learning, and computational modeling is beginning to address these challenges. These technologies facilitate the systematic prediction of compound–target interactions, and the identification of existing drugs with polypharmacological dual targeting potential [16].

Benzoxazinones have attracted attention due to their broad‐spectrum biological activities, including anticancer, α‐chymotrypsin antagonist, complement protein one receptor blocker, anti‐cathepsin G, an inhibitor of human leukocyte elastase, anti‐human coronavirus, antibacterial, antifungal, antiphlogistic [17, 18, 19, 20, 21, 22, 23]. Drugs CX‐614, efavirenz, and cetilistat contain benzoxazinone functionality in their molecular structures and have been developed for treating Parkinson's and Alzheimer's disease, AIDS, and obesity, respectively [24]. Therefore, we hypothesized that incorporating benzoxazinones into the therapeutic portfolio for HCC, particularly within the framework of molecular‐targeted therapy and polypharmacology, holds promise for developing comprehensive and effective treatment strategies.

This study aimed to systematically evaluate benzoxazinone derivatives as promising multi‐target therapeutic candidates through an integrative polypharmacological approach. Specifically, we sought to determine whether these derivatives could effectively modulate multiple oncogenic targets pivotal to HCC pathogenesis using advanced computational techniques, including network pharmacology and molecular docking. Additionally, we aimed to identify the most potent benzoxazinone‐based compounds with favorable pharmacokinetic and toxicity profiles via ADMET screening. Furthermore, we assessed their therapeutic efficacy and safety in comparison to existing treatments through mechanistic studies, including in vitro cytotoxicity assays, western blot analysis, and in vivo evaluations using PLC/PRF/5 tumor‐bearing and HCC patient‐derived tumor xenograft (PDTX) mouse models. To accomplish these objectives, we computationally designed and screened a focused library of benzoxazinone derivatives, experimentally validated their polypharmacological activities, and systematically identified the lead compound (ZAK‐I‐57) as a viable candidate for advanced therapeutic development.

## 2 Results

### 2.1 Benzoxazinone Derivatives: Synthesis, Characterization and Electronic Profiling Unveiled by DFT Studies

In this study, a series of benzoxazinone derivatives, designated as ZAK‐I‐55, ZAK‐I‐57, ZAK‐I‐64, ZAK‐I‐68, ZAK‐I‐87, ZAK‐I‐90, ZAK‐I‐93, and ZAK‐I‐97, were synthesized via the reaction of substituted 2‐aminobenzoic acids (**1**) with substituted benzoyl chlorides (**2** and **3**) (Figure S1). The synthesized compounds were characterized using FT‐IR, ^1^H‐NMR, ^13^C‐NMR, and mass spectrometry (please refer to Supporting Information Sections 1.2.1–1.2.8). The FT‐IR analysis exhibited two strong absorption bands in the ranges 1740–1780 cm^−1^ and 1619–1665 cm^−1^. For ZAK‐I‐93 and ZAK‐I‐97, strong absorption bands in the ranges 978 cm^−1^ and 969 cm^−1^, respectively, were attributed to the out‐of‐plane bending vibration of the C–H bond in *E*‐ethylene. Notably, the characteristic chemical shift (*δ*) values for the olefinic moiety in ZAK‐I‐93 and ZAK‐I‐97 were distinctly observed, and their trans‐geometry was confirmed with the presence of two doublets along with large coupling constants (*J* = 16.4 and 16.3 Hz, respectively). The structures of the synthesized benzoxazinones (ZAK‐I‐55, ZAK‐I‐57, ZAK‐I‐64, ZAK‐I‐68, ZAK‐I‐87, ZAK‐I‐90, ZAK‐I‐93, and ZAK‐I‐97) were confirmed using ^1^H‐NMR and ^13^C NMR analysis.

**FIGURE 1:**
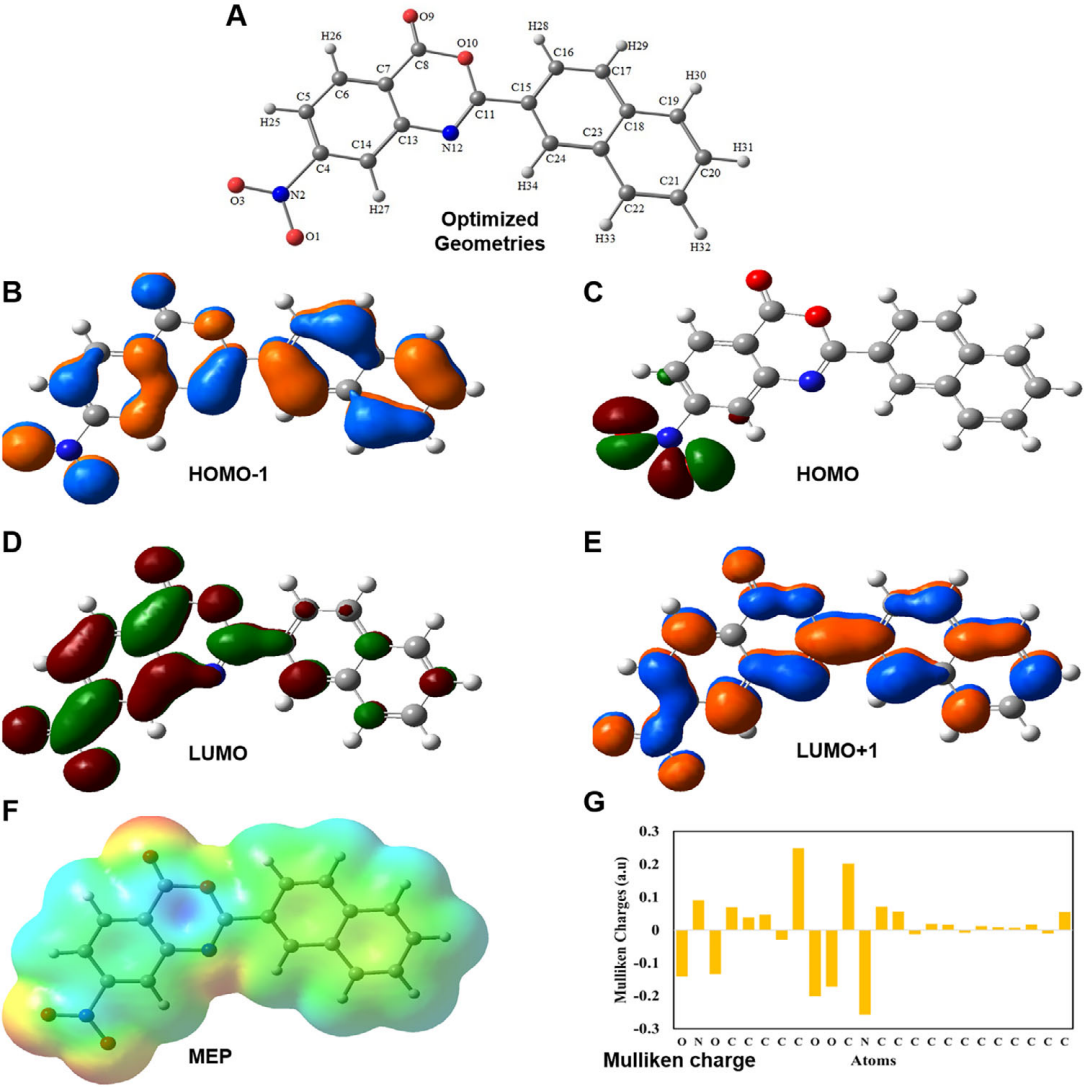
DFT study. (A) Optimized geometry, (B) HOMO‐1, (C) HOMO, (D) LUMO, and (E) LUMO+1 of ZAK‐I‐57. HOMO‐1 orbitals are located all over ZAK‐I‐57. The HOMO orbitals were located on the nitro group and core of ZAK‐I‐57. The LUMO and LUMO+1 orbitals were located all over ZAK‐I‐57. (F) MEP mapping. Green, orange, blue, red, and yellow on the MEP surfaces indicate the order of magnitude of the electrostatic potential throughout the structures. The colors were arranged in increasing order: red > orange > yellow > green > blue. (G) Mulliken charges of ZAK‐I‐57.

Comprehensive DFT analysis of benzoxazinone derivatives further provides critical insights into their geometric, electronic, and reactivity characteristics, which are essential for their potential applications in biological systems. The global reactivity indices analysis (Table S1) identifies ZAK‐I‐97 and ZAK‐I‐93 as highly electrophilic and chemically soft, making them well‐suited for electron‐rich environments. In contrast, ZAK‐I‐57 and ZAK‐I‐64 have emerged as potent electron donors. The optimized geometries highlighted the influence of various substituents on the benzoxazinone core (Tables S2–S9). For instance, the incorporation of a naphthyl group in ZAK‐I‐57 and a hydroxyl group in ZAK‐I‐68 introduced significant electronic effects, which likely enhanced the overall reactivity of these molecules (Figure 1A and Figure S2). Frontier molecular orbital (FMO) analysis revealed that ZAK‐I‐57 exhibited the smallest HOMO‐LUMO gap (Δ*E* = 2.26 eV) (Table S10, Figure 1B–E, and Figure S3), indicating high reactivity and a propensity for electron transfer processes. These properties are crucial for facilitating interactions with biological targets [25], such as charge transfer or redox modulation in anticancer applications. The natural bond orbital (NBO) analysis further emphasizes the extensive π→π* interactions in ZAK‐I‐87, with particularly high stabilization energies, reflecting robust electron delocalization and contributing to its structural stability (Tables S11–S15). Molecular electrostatic potential (MEP) mapping (Figure 1F and Figure S4) and Mulliken charge analysis (Figure 1G and Figure S5) provided additional layers of understanding, elucidating the electrostatic interactions and charge distributions that define the reactivity patterns of these derivatives. Taken together, the DFT study provides theoretical support for the potential of ZAK‐I‐57 and ZAK‐I‐87 as candidates for further biological evaluation, particularly in the context of anticancer applications such as HCC.

### 2.2 Benzoxazinone Derivatives Exhibit Promising Drug‐Like Properties and Multi‐Targeted Anti‐HCC Potential Through Systems Pharmacology

The physicochemical, pharmacokinetic, drug‐likeness, and medicinal chemistry properties of benzoxazinone derivatives were predicted using SwissADME [26] (Tables S16–S18). All compounds adhered to Lipinski's rule of five, exhibited high GI absorption, and showed no BBB permeability [27, 28, 29]. Notably, most derivatives inhibited CYP1A2 and CYP2C9, with variations in CYP2C19 inhibition. Synthetic accessibility scores (2.86–3.31) indicate ease of synthesis, and no PAINS alerts were detected [30]. While Brenk alerts suggested potential specificity [31, 32]. Overall, these derivatives demonstrated favorable DMPK and ADMET profiles, supporting their potential for further development.

A total of 265 potential protein targets of benzoxazinone derivatives were identified using SwissTargetPrediction (Figure S6A) [33]. Note that 564 HCC‐related targets were retrieved from OncoDB, HCC, and Liverome databases (Figure S6A) [34, 35, 36] and 50 intersecting targets were identified using VENNY 2.1.0 [37] (Figure S6B and Table S19). Subsequently, STRING [38] analysis of these intersecting targets revealed a PPI network comprising 50 nodes and 390 edges, with an average node degree of 15.6 (Figure S6C). The PPI network was further analyzed in Cytoscape software (version 3.9.0) [39] as shown in Figure S6D, identifying 23 key nodes (DC > 31.83) as potential anti‐HCC core targets (Figures S6E and S7A). The top six anti‐HCC core targets (c‐Myc, ESR1, EGFR, HSP90AA1, CCND1, and ERBB2) were validated for differential expression in LIHC samples, showing a strong correlation with HCC progression (Figure S7B). Due to their critical roles in oncogenesis, these targets were selected for molecular docking to evaluate their interactions with benzoxazinone derivatives.

A compound‐target network of eight benzoxazinone derivatives with 50 anti‐HCC targets was constructed using Cytoscape (Figure S8A), revealing 60 nodes, 137 edges, four diameters, two radii, 1.199 heterogeneity, 0.077 density, and an average path length of 2.696. Nodes, color‐coded from red (highest degree) to green (lowest degree), represent target connectivity, while edges indicate compound‐target interactions. A hub network was further constructed between eight benzoxazinone derivatives and 23 anti‐HCC core targets (Figure S8B). Degree centrality (DC) analysis identified five key derivatives—ZAK‐I‐57, ZAK‐I‐64, ZAK‐I‐68, ZAK‐I‐87, and ZAK‐I‐93—exceeding the threshold (average DC > 8.875) and interacting with more than eight core targets (Figure S8C). The hub network confirms the multi‐targeting nature of benzoxazinone derivatives, where a single compound modulates multiple anti‐HCC targets, and multiple compounds engage the same oncogenic target, suggesting a synergistic inhibitory effect on HCC progression.

Gene ontology (GO) enrichment analysis of 50 anti‐HCC core targets identified 97 biological processes (BP), 31 cellular components (CC), and 46 molecular functions (MF) (*p* ≤ 0.05) [40]. The top enriched BP terms included protein phosphorylation, negative regulation of the apoptotic process, cytokine‐mediated signaling pathway, positive regulation of protein kinase B signaling, and MAPK cascade (Figure S9). The enriched CC terms localized anti‐HCC targets to the cytoplasm, cytosol, plasma membrane, extracellular exosomes, and nucleoplasm, while MF enrichment predominantly involved protein binding, ATP binding, protein serine/threonine/tyrosine kinase activity, protein kinase activity, and protein kinase binding, reinforcing the therapeutic relevance of benzoxazinone derivatives in modulating key MF and cellular processes involved in HCC progression. Kyoto Encyclopedia of Genes and Genomes (KEGG) pathway analysis further elucidated the mechanistic involvement of benzoxazinone derivatives in HCC therapy, identifying six key pathways: cancer‐associated signaling (23 targets), proteoglycans in cancer (16), ErbB signaling (9), PI3K‐Akt pathway (14), EGFR tyrosine kinase inhibitor (TKI) resistance (8), and VEGF signaling (7) (Figure S10). These results highlight the broad involvement of anti‐HCC targets in multiple oncogenic pathways, suggesting that benzoxazinone derivatives exert their therapeutic effects through a multi‐targeted mechanism, making them promising candidates for HCC treatment.

**FIGURE 2:**
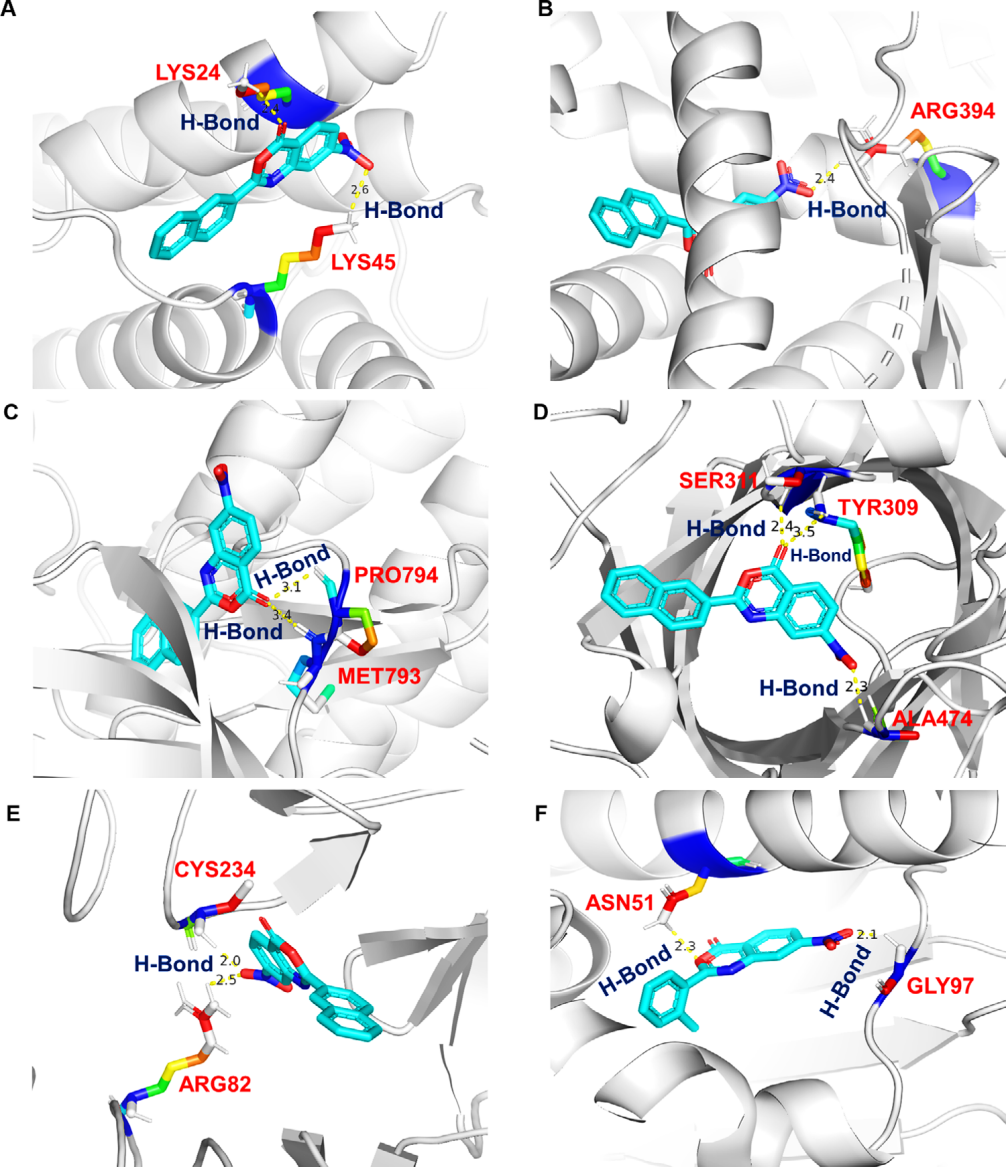
Molecular docking interactions of ZAK‐I‐57 and ZAK‐I‐64 with key oncogenic proteins. ZAK‐I‐57 binds to (A) c‐Myc (B) ESR1, (C) EGFR, (D) CCND1, and (E) ERBB2. ZAK‐I‐64 binds (F) HSP90AA1.

Molecular docking of five key active benzoxazinone derivatives with six anti‐HCC core targets (c‐Myc, ESR1, EGFR, HSP90AA1, CCND1, and ERBB2) was performed and the binding affinity are summarized in Table S20. A lower binding energy signifies stronger ligand‐receptor interactions, indicating high binding affinity. Docking results revealed that all benzoxazinone derivatives exhibited strong binding affinities toward the six core targets, supporting their multi‐targeted potential in HCC therapy. Among these, ZAK‐I‐57 (−6.6 kcal/mol) and ZAK‐I‐68 (−6.5 kcal/mol) showed the strongest binding to c‐Myc, while ZAK‐I‐57 (−9.4 kcal/mol) and ZAK‐I‐64 (−9.0 kcal/mol) had the highest affinity for ESR1. For EGFR, ZAK‐I‐57 (−8.0 kcal/mol) and ZAK‐I‐68 (−7.4 kcal/mol) exhibited the lowest binding energies. ZAK‐I‐64 (−10.1 kcal/mol) and ZAK‐I‐87 (−9.8 kcal/mol) showed the strongest binding to HSP90AA1. For CCND1, ZAK‐I‐57 demonstrated the highest binding affinity with an energy score of ‐8.0 kcal/mol, while ZAK‐I‐64 and ZAK‐I‐87 showed identical binding affinities of −7.6 kcal/mol each. Lastly, ZAK‐I‐57 (−6.3 kcal/mol) and ZAK‐I‐68 (−5.9 kcal/mol) exhibited strong affinity for ERBB2. The docked ligand‐receptor complexes with the best binding affinities are depicted in Figure 2A–F. ZAK‐I‐57 established hydrogen bonds with c‐Myc (LYS24: 2.4 Å, LYS45: 2.6 Å), ESR1 (ARG394: 2.4 Å), EGFR (MET793: 3.4 Å, PRO794: 3.16 Å), CCND1 (TYR309: 3.5 Å, SER311: 2.4 Å, ALA474: 2.3 Å), and ERBB2 (ARG82: 2.5 Å, CYS234: 2.0 Å), engaging critical residues involved in oncogenic signaling and transcriptional regulation. ZAK‐I‐64 exhibited strong hydrogen bonding interactions with HSP90AA1 (ASN51: 2.3 Å, GLY97: 2.1 Å), targeting its chaperone function essential for protein stability in cancer cells. Thus, the docking results indicate that benzoxazinone derivatives, such as ZAK‐I‐57, ZAK‐I‐64, and ZAK‐I‐68, are the most important and significant compounds that may be effective in suppressing oncogene proteins implicated in HCC.

### 2.3 Benzoxazinone Derivatives Exhibit Selective Cytotoxicity and Modulated Multiple Oncogenic Targets

**FIGURE 3:**
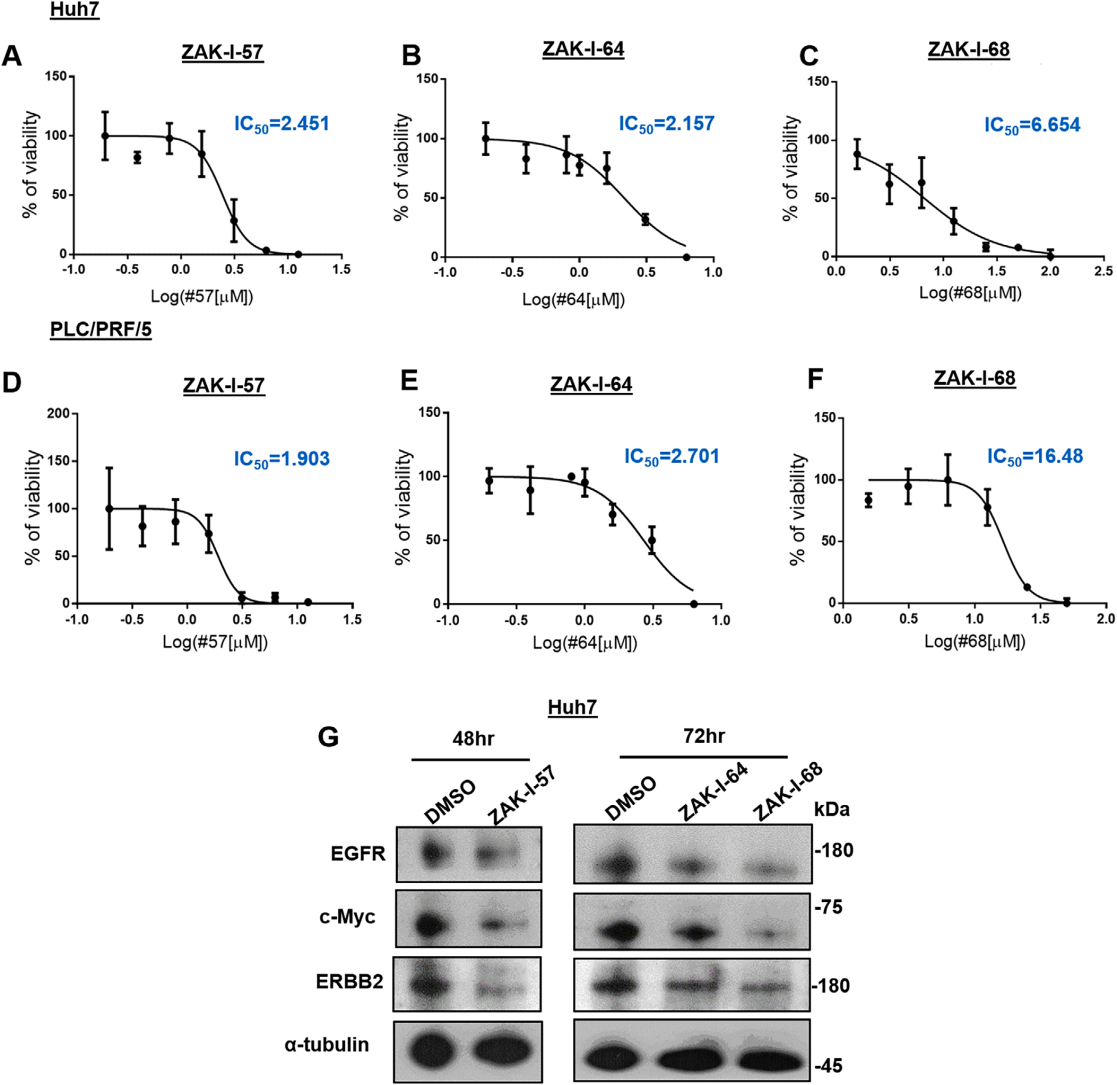
In vitro evaluation of benzoxazinone derivatives in Huh7 and PLC/PRF/5 cells. (A–F) Cell viability percentage of Huh7 and PLC/PRF/5 cell lines treated with ZAK‐I‐57 (24 h), ZAK‐I‐64 (48 h), and ZAK‐I‐68 (48 h). (G) Western blotting results. EGFR, c‐Myc, and ERBB2 expression levels after treatment with ZAK‐I‐57 (48 h), ZAK‐I‐64 (72 h), and ZAK‐I‐68 (72 h).

Three benzoxazinone derivatives (ZAK‐I‐57, ZAK‐I‐64, and ZAK‐I‐68) exhibited the most promising results in system pharmacology and molecular docking studies, prompting further investigation of their cytotoxic activity in Huh7 and PLC/PRF/5 cell lines. As shown in Figure 3A–F, a concentration‐dependent increase in cytotoxicity was observed for all three compounds in both cell lines. Notably, ZAK‐I‐57 demonstrated the highest toxicity after 24 h of treatment (Figure 3A,D), while ZAK‐I‐64 and ZAK‐I‐68 exerted comparable cytotoxic effects after 48 h (Figure 3B,C,E,F). Following these promising results, ZAK‐I‐57 was further evaluated against MIHA, an immortalized normal liver cell line. ZAK‐I‐57 exhibited negligible cytotoxicity (IC_50_ > 100 µM at 24 h and 48 h; Figure S11A,B), underscoring its selectivity and therapeutic potential. Western blot analysis revealed significant downregulation of EGFR, c‐Myc, and ERBB2 after 48 h treatment with ZAK‐I‐57 (Figure 3G), whereas ZAK‐I‐64 and ZAK‐I‐68 exhibited no appreciable effect at this time point. However, prolonged exposure to 72 h resulted in significant downregulation of these targets by both compounds, with ZAK‐I‐68 demonstrating greater efficacy than ZAK‐I‐64. These results, in conjunction with our network pharmacology and molecular docking data, substantiate that ZAK‐I‐57, ZAK‐I‐64, and ZAK‐I‐68 effectively modulate multiple HCC‐related targets.

### 2.4 ZAK‐I‐57 Inhibits Tumor Growth by Suppressing EGFR and c‐Myc Expressions in PLC/PRF/5 Tumor‐Bearing Mice

**FIGURE 4:**
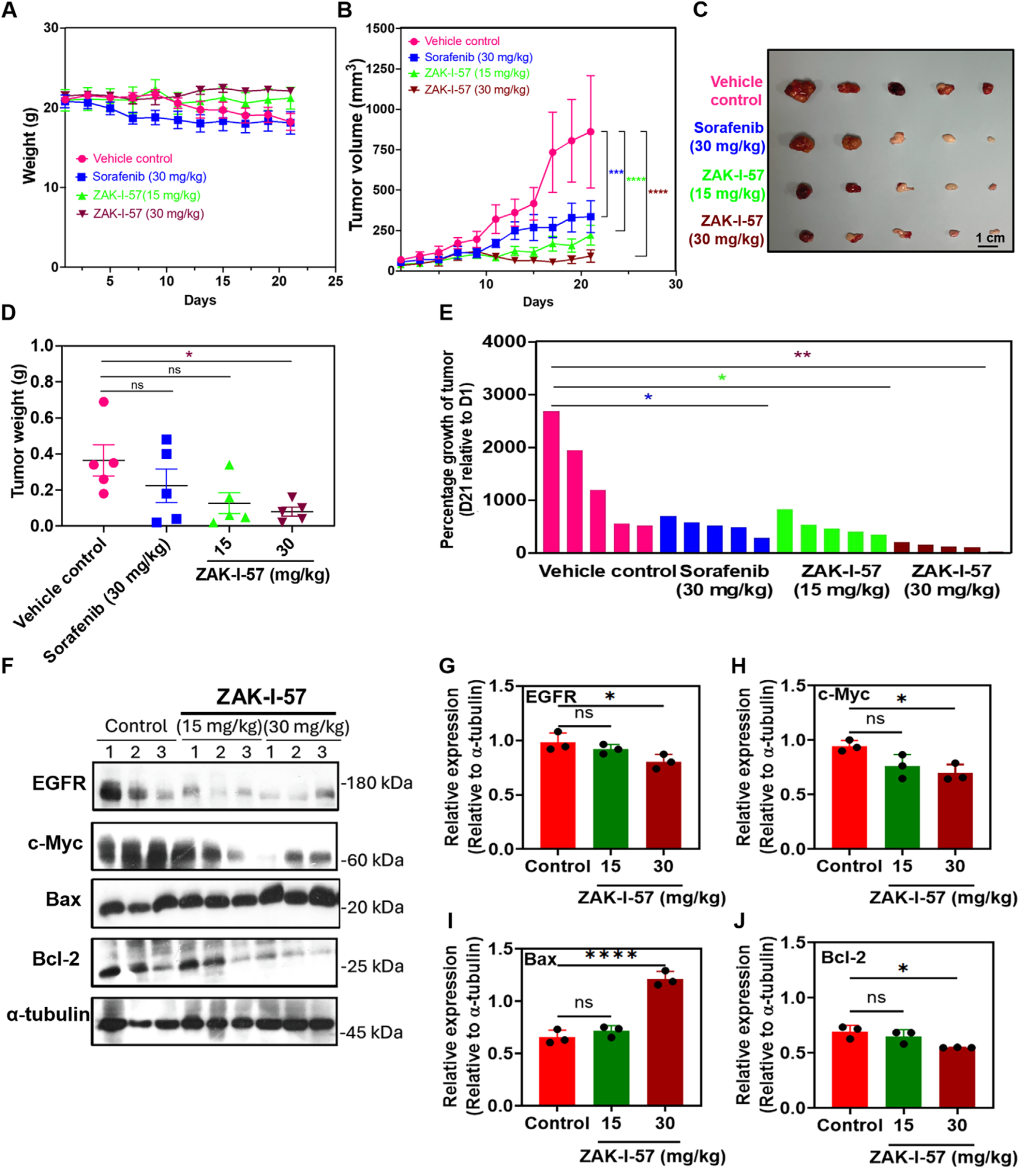
Antitumor efficacy of ZAK‐I‐57 in PLC/PRF/5 tumor‐bearing mice. (A) Body weight change curves of the mice (statistical analysis: *p* > 0.05 [control vs. sorafenib at 30 mg/kg; control vs. ZAK‐I‐57 at 15 mg/kg; control vs. ZAK‐I‐57 at 30 mg/kg]). (B and C) Tumor volume change curves (statistical analysis: ***p = 0.0004 [control vs. sorafenib at 30 mg/kg]; ****p < 0.0001 [control vs. ZAK‐I‐57 at 15 mg/kg and 30 mg/kg]) and photographs of tumors, respectively. Scale bar = 1 cm. (D) Tumor weight change of the mice (statistical analysis: ns = *p* = 0.3935 [control vs. sorafenib at 30 mg/kg]; ns = *p* = 0.0776 [control vs. ZAK‐I‐57 at 15 mg/kg]; *p = 0.0320 [control vs. ZAK‐I‐57 at 30 mg/kg]). (E) Percentage of tumor growth inhibition (*n* = 5 per group) (statistical analysis: *p = 0.0241 [control vs. sorafenib at 30 mg/kg]; *p = 0.0308 [control vs. ZAK‐I‐57 at 15 mg/kg]; **p = 0.0022 [control vs. ZAK‐I‐57 at 30 mg/kg]). (F) western blotting results for three representative PLC/PRF/5 tumor‐bearing mice from each group. Relative quantitative expression levels of (G) EGFR (ns = *p* = 0.4777; *p = 0.0339), (H) c‐Myc (ns = *p* = 0.0609; *p = 0.0195), (I) Bax (ns = *p* = 0.4673; ****p <0.0001), and (J) Bcl‐2 (ns = *p* = 0.4757; *p = 0.0180) in PLC/PRF/5 tumor‐bearing mice treated with ZAK‐I‐57 (15 mg/kg and 30 mg/kg) compared to the control.

Motivated by its exceptional in vitro performance, we further evaluated the antitumor efficacy of ZAK‐I‐57 in PLC/PRF/5 tumor‐bearing mice and compared it with that of the standard drug, that is, sorafenib. The mice were divided into four groups: vehicle control, sorafenib (30 mg/kg), ZAK‐I‐57 (15 mg/kg), and ZAK‐I‐57 (30 mg/kg), with five mice per group. Key metrics, including body weight, tumor volume, and tumor weight, were meticulously measured and assessed throughout the study. The results showed no significant changes (*p* > 0.05) in the body weight of the mice treated with ZAK‐I‐57 (15 and 30 mg/kg) for 20 days (Figure 4A). In contrast, the vehicle control and sorafenib groups exhibited only slight variation in body weight. Notably, the groups treated with ZAK‐I‐57 displayed a marked reduction in tumor volume and weight compared to the vehicle control and sorafenib‐treated groups (Figure 4B–D). Furthermore, the tumor growth percentage results demonstrated that ZAK‐I‐57 significantly inhibited tumor growth at concentrations of 15 and 30 mg/kg, demonstrating superior efficacy compared to sorafenib at 30 mg/kg (Figure 4E). Western blot analysis showed that ZAK‐I‐57 at 30 mg/kg significantly attenuated the expression of key oncogenic proteins, including EGFR, c‐Myc, and Bcl‐2, compared to the control group (Figure 4F–J). Notably, this concentration also induced an increase in the expression of Bax, a pro‐apoptotic protein, indicating a dual mechanism of action of the compound. Hence, the significant downregulation of oncogenic proteins and upregulation of pro‐apoptotic proteins underscores ZAK‐I‐57's multifaceted approach to inhibiting HCC progression, making it a promising therapeutic candidate.

### 2.5 ZAK‐I‐57 Exhibits Potent Antitumor Efficacy in PDTX Mouse Model by Significantly Suppressing the Expression of EGFR and c‐Myc

**FIGURE 5:**
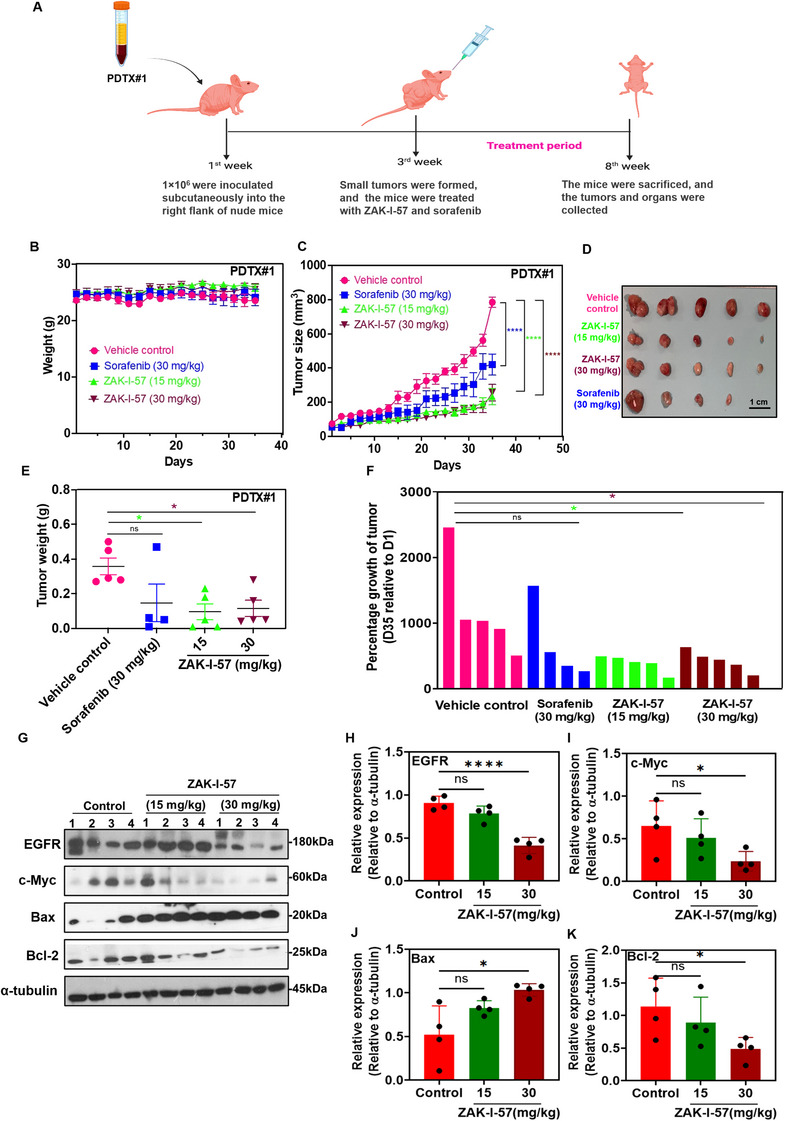
Antitumor efficacy of ZAK‐I‐57 in PDTX mouse model. (**A**) Schematic representation of the PDTX mouse model (*n* = 5 per group) (created in BioRender.com). (**B**) The body weight change curve of the mice (statistical analysis: *p* > 0.05 [control vs. sorafenib at 30 mg/kg; control vs. ZAK‐I‐57 at 15 mg/kg; control vs. ZAK‐I‐57 at 30 mg/kg]). (**C** and **D**) Tumor volume change curves (statistical analysis: ****p < 0.0001 [control vs. sorafenib at 30 mg/kg; control vs. ZAK‐I‐57 at 15 mg/kg; control vs. ZAK‐I‐57 at 30 mg/kg]) and photographs of tumors, respectively. Scale bar = 1 cm. (**E**) Tumor weight change of the mice (statistical analysis: ns = 0.0856 [control vs. sorafenib at 30 mg/kg]; *p = 0.0204 [control vs. ZAK‐I‐57 at 15 mg/kg]; *p = 0.0324 [control vs. ZAK‐I‐57 at 30 mg/kg]). (**F**) Percentage of tumor growth inhibition (statistical analysis: ns = *p* = 0.3062 [control vs. sorafenib at 30 mg/kg]; *p = 0.0218 [control vs. ZAK‐I‐57 at 15 mg/kg]; *p = 0.0273 [control vs. ZAK‐I‐57 at 30 mg/kg]). (**G**) Western blotting results for four representative PDTX mouse models from each group. Relative quantitative expression levels of (**H**) EGFR (ns = *p* = 0.1267; ****p <0.0001), (**I**) c‐Myc (ns = *p* = 0.6023; *p = 0.0496), (**J**) Bax (ns = *p* = 0.1040; *p = 0.0103), and (**K**) Bcl‐2 (ns = *p* = 0.5331; *p = 0.0491) in PDTX mouse models.

We extended our investigation to evaluate its potential in the PDTX (PDTX#1)‐derived HCC model [10] (Figure 5A), comparing it to the standard drug sorafenib. Mice were divided as described in Section 2.4, with each group comprising five mice, except for the sorafenib group, which consisted of four mice. Notably, no significant difference (*p* > 0.05) was observed between the ZAK‐I‐57 treatment groups and control group, indicating a lack of significant toxicity. Slight variations in the weight of the vehicle control and sorafenib groups were also observed compared to the ZAK‐I‐57‐treated groups at both dosages (Figure 5B). Figure 5C,D demonstrates that ZAK‐I‐57 significantly inhibited tumor progression in a dose‐dependent manner, with a significant reduction in tumor weight at the dose of 30 mg/kg (Figure 5E). Figure 5F illustrates that the percentage of tumor growth was markedly diminished in the groups treated with ZAK‐I‐57 (15 mg/kg and 30 mg/kg) compared to that in the vehicle control group. Western blot analysis presented in Figure 5G–K further demonstrates that at a dose of 30 mg/kg, ZAK‐I‐57 significantly (*p* < 0.05) downregulated EGFR and c‐Myc, two pivotal oncogenic drivers in HCC, indicating strong inhibition of proliferative signaling pathways. Simultaneously, ZAK‐I‐57 demonstrated a potent pro‐apoptotic effect by significantly upregulating Bax and downregulating Bcl‐2 at 30 mg/kg dose. This dual modulation not only curtails tumor growth but actively promotes tumor cell death, showcasing ZAK‐I‐57's comprehensive anti‐tumor capabilities.

### 2.6 ZAK‐I‐57 Demonstrates Antitumor Efficacy by Inducing Necrosis and Suppressing Proliferation in PLC/PRF/5 Tumor‐Bearing and PDTX Mouse Models, Surpassing Sorafenib

**FIGURE 6:**
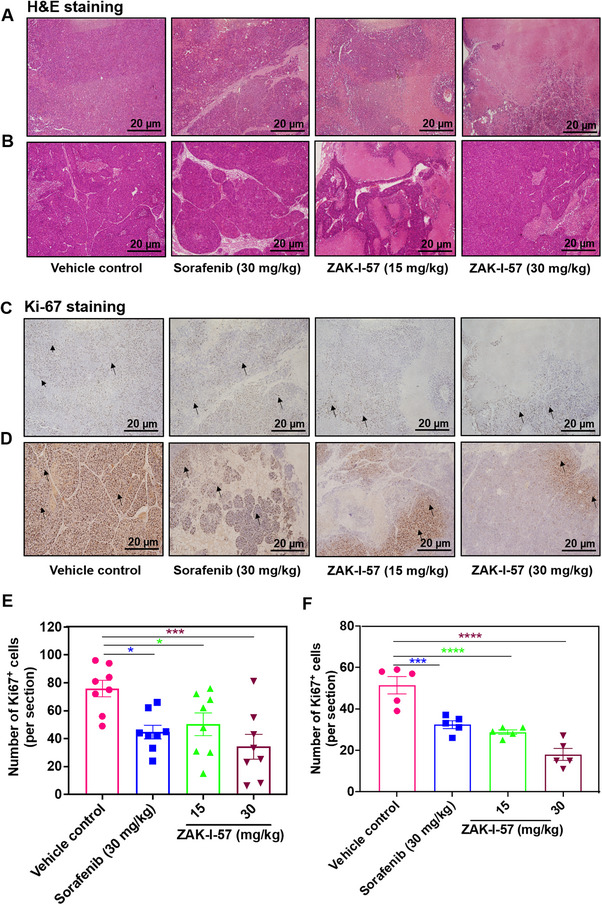
Effect of ZAK‐I‐57 on morphological and proliferative assessments on tumors in both PLC/PRF/5 tumor‐bearing and PDTX mouse models. H&E staining of (**A**) PLC/PRF/5 tumor‐bearing mouse tissues and (**B**) PDTX mouse model tissues treated with sorafenib (30 mg/kg) and ZAK‐I‐57 at two doses (15 and 30 mg/kg) compared with vehicle control. Ki‐67 staining of (**C**) PLC/PRF/5 tumor‐bearing mouse tissues and (**D**) PDTX mouse model tissues treated with sorafenib (30 mg/kg) and ZAK‐I‐57 at two doses (15 and 30 mg/kg) compared with the vehicle control. Scale bar = 20 µm. Arrows indicate Ki‐67^+^ cells. Quantitative determination of the number of Ki67^+^ stained cells in (**E**) PLC/PRF/5 tumor‐bearing mouse tissues (statistical analysis: *p = 0.0120 [control vs. sorafenib at 30 mg/kg]; *p = 0.0431 [control vs. ZAK‐I‐57 at 15 mg/kg]; ***p = 0.0008 [control vs. ZAK‐I‐57 at 30 mg/kg]) and (**F**) PDTX mouse model tissues (***p = 0.0005; ****p < 0.0001) treated with sorafenib (30 mg/kg) and ZAK‐I‐57 at two doses (15 and 30 mg/kg) compared with vehicle control, respectively.

Histopathological and proliferation analysis using hematoxylin & eosin (H&E) and Ki‐67 staining confirmed the significant antitumor effects of ZAK‐I‐57 in PLC/PRF/5 tumor‐bearing and PDTX mouse model tissues, indicating its potential as a superior therapeutic agent compared to sorafenib, as shown in Figure 6A–F. H&E staining demonstrated a marked reduction in tumor cellularity following treatment with ZAK‐I‐57 (15 and 30 mg/kg) and sorafenib (30 mg/kg) compared to the vehicle control. Notably, the 30 mg/kg dose of ZAK‐I‐57 induced substantial decrease in cell density and necrosis, paralleling the effects observed with sorafenib. In addition, ZAK‐I‐57 exhibited antiproliferative activity, as evidenced by the inhibition of Ki‐67 expression in both HCC xenograft models (Figure 6E,F).

### 2.7 ZAK‐I‐57 Showcases Excellent Biosafety Profile Across Vital Organs at Both Doses in PLC/PRF/5 Tumor‐Bearing and PDTX Mouse Models

**FIGURE 7:**
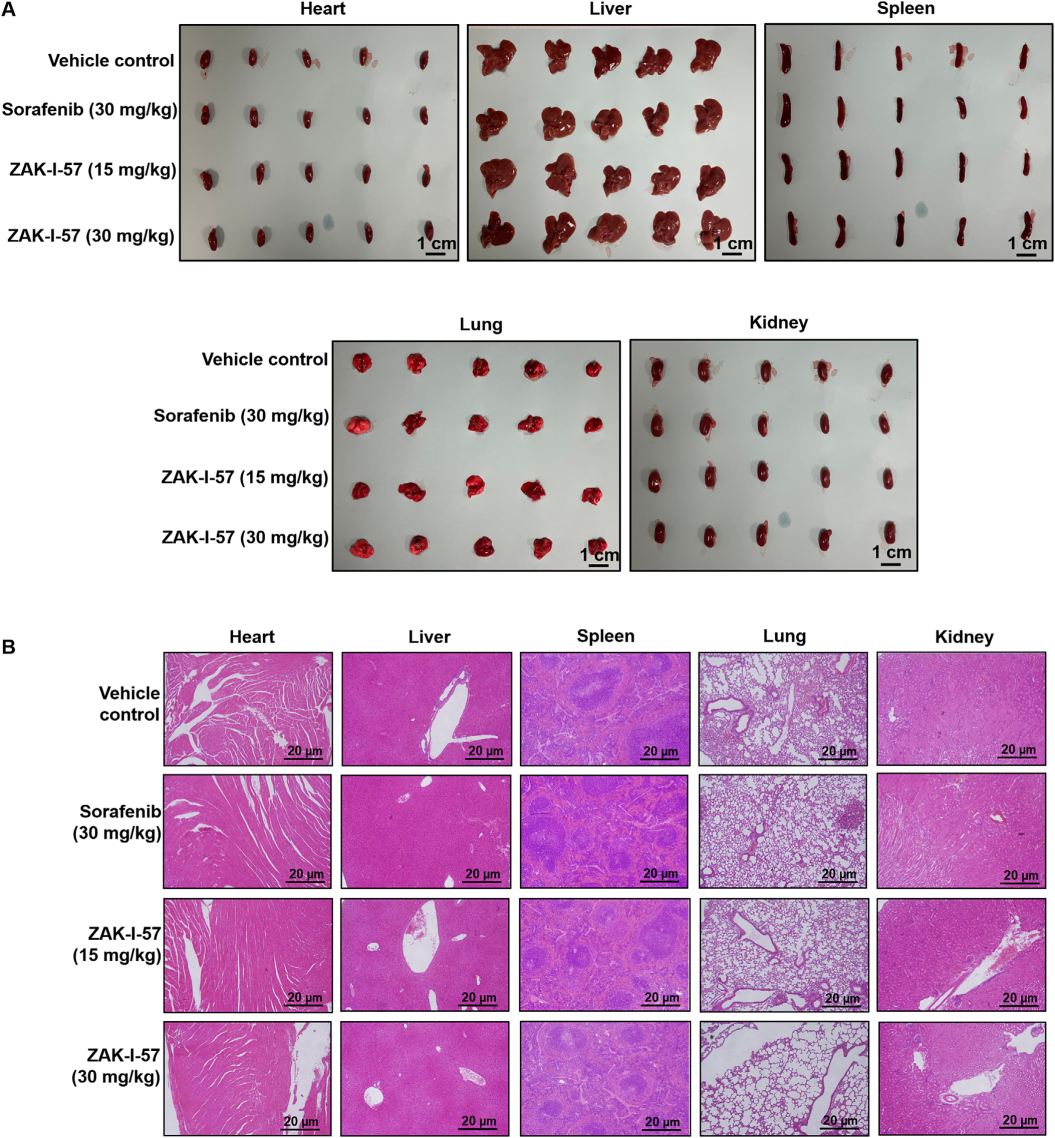
Effect of ZAK‐I‐57 on morphological and histological assessments of major organs in PLC/PRF/5 tumor‐bearing mice. (**A**) Gross anatomical assessment of vital organs (heart, liver, kidney, spleen, and lungs) from a PLC/PRF/5 tumor‐bearing mouse model administered a control vehicle, sorafenib at a therapeutic dose of 30 mg/kg, and ZAK‐I‐57 at two doses (15 and 30 mg/kg). Scale bar = 1 cm. (**B**) Histopathological compendium delineates H&E‐stained organ sections from a PLC/PRF/5 tumor‐bearing mouse model administered a control vehicle, sorafenib at a therapeutic dose of 30 mg/kg, and ZAK‐I‐57 at two doses (15 and 30 mg/kg). Scale bar = 20 µm.

Comprehensive biosafety evaluations in both PLC/PRF/5 (Figure 7) and PDTX (Figure S12) mouse models confirmed the safety of ZAK‐I‐57 as a therapeutic agent for HCC. Gross anatomical examination of vital organs including heart, liver, spleen, lungs, and kidneys across both models showed that ZAK‐I‐57 (15 and 30 mg/kg) and sorafenib (30 mg/kg) maintained tissue morphology comparable to vehicle control, indicating minimal off‐target toxicity (Figure 7A and Figure S12A). Histopathological analysis further confirmed the absence of significant pathological changes in all treatment groups. In both PLC/PRF/5 model (Figure 7B) and PDTX model (Figure S12B), no histological abnormalities were observed in heart, liver, spleen, lungs, or kidneys, reinforcing the safety of ZAK‐I‐57. These findings underscore ZAK‐I‐57's exceptional biosafety profile, positioning it as a promising therapeutic candidate for HCC.

## 3 Discussion

The high molecular heterogeneity of HCC presents a formidable challenge in therapeutic intervention [1, 2]. Existing molecular‐targeted agents, such as sorafenib and lenvatinib, have demonstrated limited efficacy due to single‐target action, acquired resistance, and dose‐limiting toxicities [41, 42, 43, 44]. To overcome these barriers, this study applies a polypharmacology‐driven strategy [45] to rationally design multi‐target benzoxazinone derivatives, particularly ZAK‐I‐57, as a superior alternative to conventional TKIs. The integrative approach, combining systems pharmacology, molecular docking, in vitro and in vivo validation, and PDTX models, provides compelling evidence of ZAK‐I‐57's potential as a next‐generation HCC therapy.

ZAK‐I‐57 exhibits structural and physicochemical attributes that offer significant pharmacological advantages over sorafenib, reinforcing its candidacy as an optimized therapeutic agent for HCC (Table S21). Its lower molecular weight (318.28 g/mol compared to 464.82 g/mol) enhances oral bioavailability and membrane permeability. The fully aromatic framework (Csp^3^ = 0.00) facilitates π–π stacking interactions, thereby strengthening target binding. Moreover, the compound's greater rigidity, characterized by only two rotatable bonds (vs. nine in sorafenib), improves binding selectivity and stability. With a balanced polar surface area (TPSA: 88.92 Å^2^), ZAK‐I‐57 maintains optimal membrane permeability while ensuring effective target interactions. The absence of hydrogen bond donors (0 compared to 3 in sorafenib) reduces excessive polarity, favoring improved pharmacokinetics. Collectively, these structural refinements highlight ZAK‐I‐57's superior drug‐like properties, bolstering its viability as a next‐generation multi‐targeted therapeutic for HCC.

Given the complex interplay of oncogenic signaling pathways in HCC, a multi‐faceted therapeutic approach is important [46]. Using systems pharmacology, we identified 50 anti‐HCC core targets, including EGFR, c‐Myc, ERBB2, ESR1, CCND1, and HSP90AA1, all of which play critical roles in tumor initiation, proliferation, angiogenesis, and resistance mechanisms [23, 47, 48, 49, 50, 51, 52, 53]. The functional enrichment analysis revealed that these targets regulate kinase signaling, apoptotic pathways, and cellular stress responses [23, 47, 48, 49, 50, 51, 52, 53], reinforcing the potential of benzoxazinone derivatives as broad‐spectrum inhibitors. KEGG pathway analysis further highlighted that these targets contribute to EGFR TKI resistance, ErbB signaling, PI3K‐Akt, and VEGF pathways, all of which are implicated in HCC aggressiveness and therapeutic resistance [54, 55, 56, 57]. Unlike single‐target therapies, ZAK‐I‐57 inhibits multiple oncogenic nodes, reducing the probability of compensatory pathway activation, a common limitation of current TKIs [58, 59].

To establish a structural basis for ZAK‐I‐57's multi‐target efficacy, molecular docking was performed against six anti‐HCC core targets. The high binding affinities of ZAK‐I‐57 toward EGFR (MET793, PRO794), c‐Myc (LYS24, LYS45), and ERBB2 (ARG82, CYS234) suggest a robust inhibitory profile. The interaction at MET793 within EGFR's ATP‐binding pocket suggests that ZAK‐I‐57 may function as a non‐ATP‐competitive inhibitor, a crucial distinction that reduces the likelihood of resistance mutations, a limitation seen with erlotinib‐resistant HCC case [60]. c‐Myc, a key transcriptional regulator in HCC, lacks a traditional druggable pocket [61], yet ZAK‐I‐57 effectively interacts with LYS24 and LYS45, suggesting a potential disruption of Myc‐Max dimerization, a critical step for oncogenic transcriptional activation [62]. Given that c‐Myc amplification is associated with poor prognosis in HCC and is a key driver of metabolic reprogramming and cell cycle progression, its inhibition represents a significant therapeutic advantage over conventional kinase inhibitors [47]. Furthermore, ZAK‐I‐57's interaction with ERBB2 at ARG82 and CYS234 suggests disruption of dimerization‐dependent activation. Unlike existing ERBB2 inhibitors, which focus on kinase domain inhibition, ZAK‐I‐57 appears to interfere with receptor dimerization and downstream oncogenic signaling, broadening its therapeutic potential [63].

Consistent with the molecular docking predictions, we further confirmed that ZAK‐I‐57 effectively suppressed expression of EGFR, c‐Myc, and ERBB2 in cell‐based model. In PLC/PRF/5 tumor‐bearing mice and PDTX mouse models, ZAK‐I‐57 treatment (30 mg/kg) resulted in substantial downregulation of EGFR, c‐Myc, and Bcl‐2, with a concurrent increase in Bax expression (Figures 4F–J and 5G–K). Bcl‐2 overexpression is a hallmark of HCC resistance to chemotherapy, as it inhibits mitochondrial outer membrane permeabilization (MOMP), preventing cytochrome c release and apoptotic cascade activation [64]. The observed upregulation of Bax and concomitant downregulation of Bcl‐2 provides strong evidence for the activation of the intrinsic apoptotic pathway, a crucial mechanism often suppressed in HCC [64, 65]. These findings suggest that ZAK‐I‐57 exerts both direct oncogenic inhibition and apoptotic reprogramming, reinforcing its potential as a comprehensive therapeutic strategy.

The in vitro cytotoxicity results demonstrated that ZAK‐I‐57 exhibits potent and selective cytotoxicity against HCC cells, with IC_50_ values lower than sorafenib [66] and artesunate [67]. More importantly, ZAK‐I‐57 displayed minimal cytotoxicity in normal hepatocytes (MIHA cells, IC_50_ > 100 µM), confirming its high selectivity. This selective cytotoxicity profile is crucial for reducing off‐target toxicities, a major limitation of first‐generation TKIs. Both PLC/PRF/5 tumor‐bearing mice and PDTX mouse models demonstrated dose‐dependent tumor suppression, with ZAK‐I‐57 at 30 mg/kg outperforming sorafenib in reducing tumor volume and weight. This superior efficacy is attributed to its simultaneous inhibition of multiple oncogenic drivers and apoptotic reprogramming. The reduction in Ki‐67‐positive cells further confirmed its strong antiproliferative effects, with the 30 mg/kg dose inducing significantly greater Ki‐67 suppression than sorafenib. A major limitation of current HCC therapies is their systemic toxicity, leading to hepatic dysfunction, cardiovascular complications, and renal impairment [68]. Histopathological analysis of vital organs (heart, liver, spleen, lungs, kidneys) revealed no significant toxicological abnormalities in ZAK‐I‐57‐treated mice, in stark contrast to sorafenib's reported hepatotoxicity. The absence of weight loss, organ damage, or significant biochemical alterations suggests that ZAK‐I‐57 possesses a superior therapeutic window compared to existing TKIs.

**FIGURE 8:**
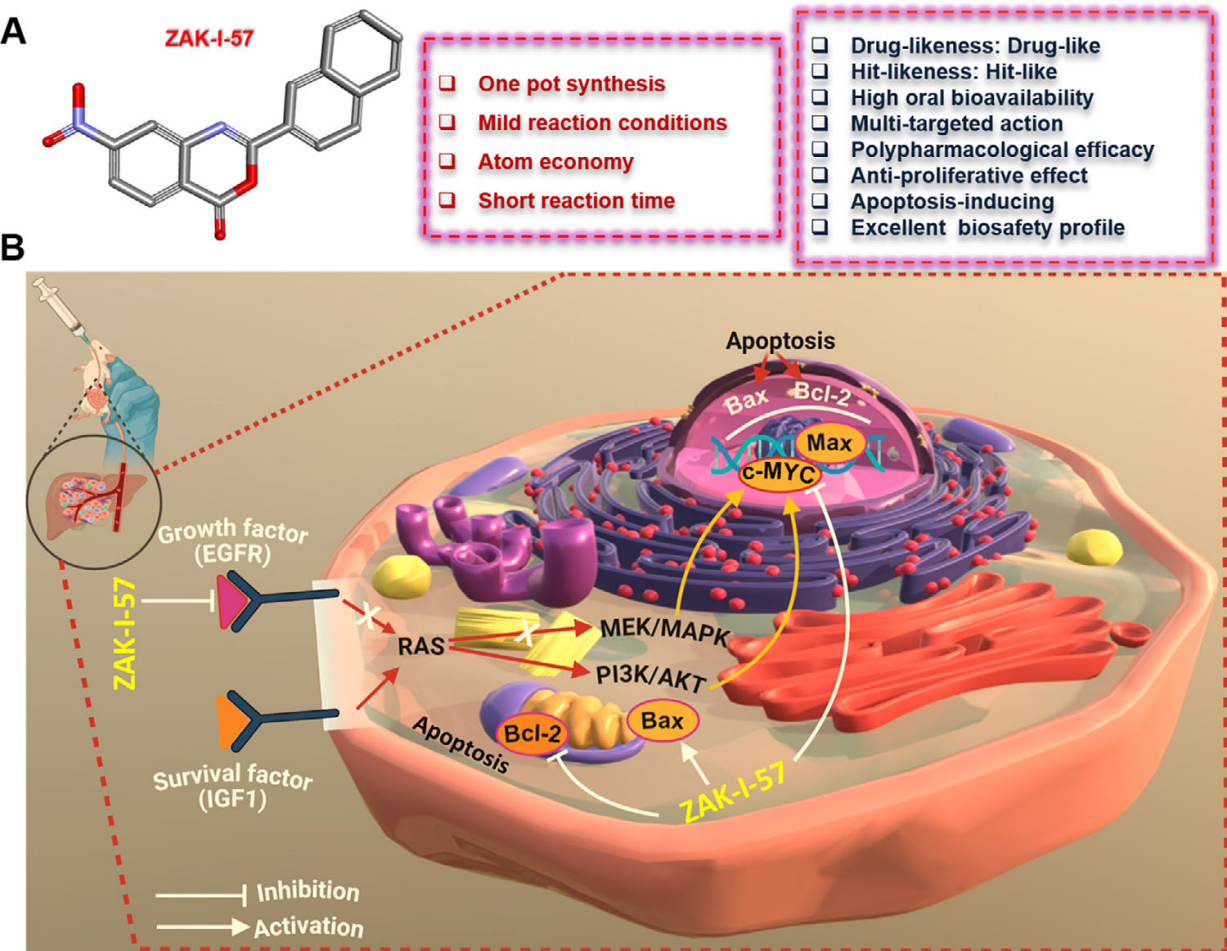
Schematic diagram of the molecular mechanisms of ZAK‐I‐57 in the treatment of HCC. (**A**) Structural representation of ZAK‐I‐57 and its core characteristics. The key pharmacological attributes of the compounds are highlighted in the upper right, showing drug‐likeness, hit‐likeness, high oral bioavailability, and other pharmacological properties. (**B**) Mechanistic pathway of ZAK‐I‐57: ZAK‐I‐57 inhibits EGFR, suppresses the MEK/MAPK signaling pathway, downregulates Bcl‐2, and upregulates Bax in the mitochondria, thereby promoting apoptosis. Additionally, it inhibits the oncogenic transcription factor c‐Myc, thereby enhancing its antiproliferative and pro‐apoptotic effects (adapted from Sketchfab.com under CC BY 4.0; modified and assembled using BioRender.com.).

In summary, ZAK‐I‐57 is established as a highly potent multi‐targeted therapeutic with superior efficacy, selectivity, and safety over existing HCC treatments. By concurrently disrupting key oncogenic pathways, modulating apoptotic regulators, and demonstrating robust tumor suppression, it presents a significant advancement in molecular‐targeted therapy (Figure 8A,B). Its polypharmacological design, strong translational potential, and excellent safety profile reinforce its viability for clinical development. While ZAK‐I‐57 demonstrates robust multi‐target efficacy and a favorable safety profile, certain limitations remain. Potential adaptive resistance mechanisms require further investigation to evaluate long‐term therapeutic effectiveness. Additionally, comprehensive pharmacokinetic and biodistribution studies are needed to elucidate its metabolic stability and clearance. Future studies will also focus on exploring combinatorial strategies with existing HCC therapies to enhance efficacy and mitigate potential resistance, supporting its clinical translation.

## 4 Materials and Methods

For detailed information on the methodologies employed for synthesis, DFT studies, and computational systems pharmacology analysis, please refer to the Supporting Information.

### 4.1 Cell Culture

Huh7 and PLC/PRF/5 (CRL‐8024) cells were purchased from Thermo Fisher Scientific and the ATCC, respectively. MIHA was kindly provided by Dr. J.R. Chowdhury, Albert Einstein College of Medicine, New York [69]. The cell lines were cultured in Dulbecco's modified Eagle medium (DMEM, Gibco, MA, USA) supplemented with 10% fetal bovine serum (FBS, BI, MA, USA) and 1x penicillin‐streptomycin solution (Solarbio) at 37°C in a humid, 5% CO_2_ atmosphere [70, 71].

### 4.2 MTT Assay

Huh7 and PLC/PRF/5 cells at a density of 5 × 10^3^ cells/well were cultured in 96‐well plates and incubated at 37°C in 5% CO_2_. Both cell lines were treated with different concentrations of benzoxazinone derivatives (ZAK‐I‐57, ZAK‐I‐64, and ZAK‐I‐68). Both cell lines were incubated with ZAK‐I‐57 for 24 h and for 48 h with ZAK‐I‐64 and ZAK‐I‐68. MTT (3‐[4,5‐dimethylthiazol‐2‐yl]‐2,5‐diphenyltetrazolium bromide) solution was then added to each well and incubated for 3 h in a CO_2_ atmosphere at 37°C. Insoluble formazan crystals were dissolved by adding 100 µL of dimethyl sulfoxide (DMSO) to each well, followed by orbital agitation for 10 min. A minimum of three biological replicates were analyzed using a plate reader to determine IC_50_. The same experiment was performed with MIHA hepatocyte cells to evaluate the selectivity cytotoxicity of ZAK‐I‐57.

### 4.3 Animals

Male BALB/c nude mice (6–8 weeks old) were obtained from the Centralized Animal Facilities at the Hong Kong Polytechnic University. All animals were bred and housed under specific pathogen‐free conditions, with access to sterile food and water. Environmental conditions were strictly controlled, with temperature maintained at 23 ± 2°C, relative humidity between 30% and 70%, and a 12‐h light/dark cycle. Mice were group‐housed in accordance with institutional guidelines for recommended stocking density. The animal experiments were conducted according to institutional guidelines, and the experimental procedures were approved by the PolyU Animal Experimentation Ethics Committee (Ref. No.19‐20/57‐ABCT‐R‐STUDENT).

### 4.4 PLC/PRF/5 Tumor‐Bearing Mouse Model

PLC/PRF/5 cells (5 × 10^5^) were suspended in 100 µL PBS with Matrigel (1:1 ratio) and injected into the right back of nude mice. Small tumors (<70 mm^3^) were formed 2 weeks after cell inoculation. Mice were then randomized into the vehicle control group, ZAK‐I‐57‐treated groups (15 and 30 mg/kg), or sorafenib (30 mg/kg) groups. Mice were administered 200 µL of vehicle water and drugs by oral gavage daily for 3 weeks. Tumor size was measured twice a week, and tumor volumes were calculated using the formula [(length × width × depth)/2 mm^3^] while each mouse was weighed. At the end of the experiment, the mice were sacrificed, and the tumors and organs were collected for further experiments.

### 4.5 PDTX Mouse Model

The procedure for the establishment of PDTX#1 was previously described [10]. Tumor cells from PDTX#1 was inoculated subcutaneously on the back of nude mice. When the tumors reached approximately 1000 mm^3^, mice with the first generation of xenografts (P1) were sacrificed, and the xenografts were isolated and expanded for the second generation (P2). When P2 xenografts reached an average volume of 70 mm^3^, mice were subjected to ZAK‐I‐57 and sorafenib treatment. The development of PDTX was approved by the Institutional Review Board of the University of Hong Kong/Hospital Authority Hong Kong West Cluster (UW 17–056) and informed consents were obtained from patients.

### 4.6 Western Blot Assay

The Huh7 cells and tumor tissues were lysed on ice using the lysis buffer. Protein concentration was quantified using Bradford assay (Bio‐Rad, Hercules, CA, USA). Equivalent amounts of protein (25–50 µg) were loaded onto 10% sodium dodecyl sulfate‐polyacrylamide gel electrophoresis (SDS‐PAGE) and transferred to polyvinylidene fluoride (PVDF) membranes. Membranes were blocked with 5% non‐fat milk in Tris‐buffered saline Tween 20 (TBST) for 1 h. The blots were incubated with primary antibodies (EGFR [4267S, Cell Signaling Technology, MA, USA], c‐Myc [5605S, Cell Signaling Technology], Bax [2772S, Cell Signaling Technology], Bcl‐2 [sc‐7382, Santa Cruze, TX, USA], ERBB2 [2165S, Cell Signaling Technology], and α‐tubulin [62204, Invitrogen, MA, USA], 1:1000) at 4°C overnight. Subsequently, the membranes were washed thrice (15 min each) with TBST solution and incubated for 1 h with secondary horseradish peroxidase‐conjugated antibodies (NA934 and NA931, Cytiva, MA, USA, 1:5000). Blots were then detected using an ECL kit and photographed using a ChemiDoc Imaging System (Bio‐Rad, MA, USA). The bands were quantified using the ImageJ software (NIH, USA). The intensities of the bands for each protein sample were normalized to those of α‐tubulin (internal standard protein). Quantitative data are presented as a fold change of untreated control.

### 4.7 Immunohistochemistry (IHC) Staining Assay

Tumors were fixed in 4% formalin, embedded in paraffin, and sectioned at 5 µm thickness for IHC staining. Ki‐67 primary antibody (ab16667, Abcam, MA, USA) and anti‐mouse IgG recombinant secondary antibody (7056S, Cell Signaling Technology, MA, USA) were used for IHC staining. Hemotoxin (Invitrogen, MA, USA) was used to stain nuclei. The number of positively stained cells in Ki‐67‐stained slides was evaluated using ImageJ software (NIH, MD, USA).

### 4.8 H&E Staining Assay

H&E staining was performed on histological sections of organs (heart, spleen, kidney, liver, and lung) to examine the toxicity of ZAK‐I‐57 in PLC/PRF/5 tumor‐bearing and PDTX model mice. Briefly, the histological slides were deparaffinized using 100 % xylene (v/v). The slides were then washed several times with ethanol solutions of various concentrations (70%–100%, v/v) and immersed in hematoxylin for 5 min, followed by a quick dip in acidic alcohol. Next, the slides were immersed in Scott's tap water for 3 min and in 1 % eosin for 30 s. Finally, the slides were dehydrated using ethanol solutions of various concentrations (70%–100%, v/v) and 100% xylene (v/v) and mounted using a mounting medium.

### 4.9 Statistical Analysis

All in vitro data are presented as mean ± SD, whereas in vivo data are expressed as the mean ± SEM. Quantitative results were analyzed using one‐way analysis of variance (ANOVA). Statistical significance was set at *p* < 0.05. All statistical analyses were performed using Prism 5 software (GraphPad, CA, USA).

## Author Contributions

S.A.K. and T.K.W.L. conceived the project and designed the study. S.A.K., H.Y., F.Y., and C.N.C. performed experiments. S.A.K. and H. Y. analyzed the data. T.K.W.L. provided experimental materials, scientific suggestions, and supervised the whole study. S.A.K. and H.Y. wrote the manuscript. All authors reviewed the manuscript and discussed the work.

## Ethics Statement

*Animal experiments*: The animal experiments were conducted according to institutional guidelines, and the experimental procedures were approved by the PolyU Animal Experimentation Ethics Committee (Ref. No.19‐20/57‐ABCT‐R‐STUDENT). *PDTX establishment*: The study was approved by the Institutional Review Board of the University of Hong Kong/Hospital Authority Hong Kong West Cluster (UW 17–056) and informed consents were obtained from patients.

## Conflicts of Interest

The United States provisional patent (US 63/657,193) for Polypharmacological benzoxazinone derivatives for synergistic treatment of hepatocellular carcinoma has been filed. “Compound/Technique/Instrument/ (US 63/657,193) is patented but has no potential relevant financial or nonfinancial interests to disclose. The remaining authors declare no conflicts of interest.”

## Supporting information



Supporting Information

## Data Availability

All data are available from the corresponding authors upon request.
