# Supplementary material for: Polypharmacology‐Driven Discovery of ZAK‐I‐57: A Potent Multi‐Targeted Benzoxazinone Small Molecule for Hepatocellular Carcinoma Therapy
Source: MedComm (2020). 2025 Jul 27;6(8):e70291. doi: 10.1002/mco2.70291 (PMC12301169; doi:10.1002/mco2.70291)
Supplement: Supplementary file 1 — Supporting Information [file MCO2-6-e70291-s001.docx]

**Supporting Information**

**Polypharmacology-Driven Discovery of ZAK-I-57: A Potent Multi-Targeted Benzoxazinone Small Molecule for Hepatocellular Carcinoma Therapy**

Shakeel Ahmad Khan^#1^*, Huihai Yang^#1^, Fan Ying^1^, Chin Ngok Chu^1^,

Terence Kin Wah LEE^1,2^*

^1^Department of Applied Biology and Chemical Technology, The Hong Kong Polytechnic University, Hung Hom, Kowloon, Hong Kong SAR (P. R. China)

^2^State Key Laboratory of Chemical Biology and Drug Discovery, The Hong Kong Polytechnic University, Hung Hom, Kowloon, Hong Kong SAR (P. R. China)

^#^ Co-first authors

^*^Corresponding authors:

The address of Prof. Terence K.W. Lee is Room 805, Block Y, Department of Applied Biology and Chemical Technology, Lee Shau Kee Building, The Hong Kong Polytechnic University, Hong Kong. Tel: (852) 3400-8799; Fax: (852) 2364-9932; Email: terence.kw.lee@polyu.edu.hk. The address of Dr. Shakeel Ahmad Khan is Room 1208, Block Y, Department of Applied Biology and Chemical Technology, Lee Shau Kee Building, The Hong Kong Polytechnic University, Hong Kong. Tel: (852) 3400-8883; Fax: (852) 2364-9932; Email: [shakilahmad56@gmail.com](mailto:shakilahmad56@gmail.com)

1. Experimental

1.1. Materials

All reagents and solvents were obtained from commercial suppliers and used without further purification, unless specified otherwise.

1.2. Synthesis

In this study, a series of novel benzoxazinone derivatives, designated ZAK-I-55, ZAK-I-57, ZAK-I-64, ZAK-I-68, ZAK-I-87, ZAK-I-90, ZAK-I-93, and ZAK-I-97, were synthesized by reacting substituted 2-aminobenzoic acids (**1**) with substituted benzoyl chlorides (**2** or **3**) (Figure S1). The reaction was performed under mild conditions (10-30°C) in the presence of pyridine. Briefly, 0.91 g of 4-nitro anthranilic acid (**1**) (0.005 mol) in pyridine (20 ml) was added to the respective substituted benzoyl chlorides (**2** and **3**) (0.01 moles) under continuous stirring at 10 °C for ten minutes. The reaction mixture was then stirred at 30 °C for another half an hour. The reaction was monitored by thin-layer chromatography (TLC). After completion of the reaction, the product was poured onto ice-cold H_2_O. The resultant precipitate was filtered, washed with ice-cold water, and dried in an oven at 50 °C.

*1.2.1. 2-(4-bromophenyl)-7-nitro-4H-3,1-benzoxazin-4-one (ZAK-I-55)*

Yield: 86%; Grey solid; mp: 118 °C; IR (υ_max_, KBr, cm^−1^): 1749, 1624; ^1^H NMR (CDCl_3_, 400 MHz) δ: 8.49 (d, 1H, Ar–H, *J* = 2.1 Hz), 8.37 (d, 1H, Ar–H, *J* = 8.2 Hz), 8.24 (dd, 1H, Ar–H, *J* = 2.2, 8.6 Hz), 7.84 (t, 1H, Ar–H, *J* = 7.2 Hz), 7.78–7.65 (m, 3H, Ar–H); ^13^C NMR (CDCl_3_, 75 MHz) δ: 157.9, 155.8, 154.6, 152.5, 143.8, 134.6, 133.4, 132.6, 131.4, 129.6, 128.9, 122.4; ESI-MS: 346.02 [M−H]^−^; Anal. Calcd. For C_14_H_7_BrN_2_O_4_ (MW: 347.10 g/mol): C, 48.44%; H, 2.03%; N, 8.07%; Found: C, 48.45%; H, 2.02%; N, 8.06%.

*1.2.2. 2-(naphthalen-2-yl)-7-nitro-4H-3,1-benzoxazin-4-one (ZAK-I-57)*

Yield: 73%; Grey solid; mp: 92 °C; IR (υ_max_, KBr, cm^−1^):1751, 1625; ^1^H NMR (CDCl_3_, 400 MHz) δ: 8.56 (d,1H, Ar–H, *J* = 2.3 Hz), 8.41 (d, 1H, Ar–H, *J* = 8.0), 8.28 (dd, 1H, Ar–H, *J* = 2.1, 8.2 Hz), 8.38–7.62 (m, 7H, Ar–H); ^13^C NMR (CDCl_3_, 75 MHz) δ: 159.6, 156.4, 154.8, 152.4, 138.8, 134.6, 133.2, 132.4, 131.7, 131.5, 130.2, 129.6, 129.2, 129.0, 127.9, 125.6, 122.2, 117.4; ESI-MS: 317.12 [M−H]^−^; Anal. Calcd. For C_18_H_10_N_2_O_4_ (MW: 318.25 g/mol): C, 67.92%; H, 3.17%; N, 8.80%; Found: C, 67.94%; H, 3.14%; N, 8.81%.

*1.2.3. 2-(2-methylphenyl)-7-nitro-4H-3,1-benzoxazin-4-one (ZAK-I-64)*

Yield: 72%; Grey solid; mp: 79 °C; IR (υ_max_, KBr, cm^−1^):1751, 1619; ^1^H NMR (CDCl_3_, 400 MHz) δ: 8.49 (d, 1H, Ar–H, *J* = 2.1 Hz), 8.37 (d,1H, Ar–H, *J* = 8.2 Hz), 8.25 (dd, 1H, Ar–H, *J* = 2.1, 8.4 Hz), 7.65 (d, 1H, Ar–H, *J* = 8.1 Hz), 7.41–7.32 (m, 3H, Ar–H), 2.44 (s, 3H, CH_3_); ^13^C NMR (CDCl_3_, 75 MHz) δ: 159.9, 157.4, 154.6, 153.4, 141.2, 138.6, 131.5, 130.2, 129.2, 128.8, 128.6, 127.3, 124.7, 119.8, 20.1; ESI-MS: 281.08 [M−H]^−^; Anal. Calcd. For C_15_H_10_N_2_O_4_ (MW: 282.15 g/mol): C, 63.83%; H, 3.57%; N, 9.92%; Found: C, 63.84%; H, 3.56%; N, 9.90%.

*1.2.4. 2-(4-hydroxyphenyl)-7-nitro-4H-3,1-benzoxazin-4-one (ZAK-I-68)*

Yield: 83%; Yellow solid; mp: 90 °C; IR (υ_max_, KBr, cm^−1^): 3345,1740, 1626; ^1^H NMR (CDCl_3_, 400 MHz) δ: 8.52 (d,1H, Ar–H, *J* = 2.0 Hz), 8.39 (d,1H, Ar–H, *J* = 8.4 Hz), 8.29 (dd,1H, Ar–H, *J* = 2.0, 8.3 Hz), 7.90–7.81 (m, 2H, Ar–H), 7.07–6.99 (m, 2H, Ar–H), 4.22 (s, 1H, OH); ^13^C NMR (CDCl_3_, 75 MHz) δ:158.8, 156.9, 154.4, 154.2, 152.3, 136.6, 132.4, 130.8, 128.5, 122.6, 120.3, 118.6; ESI-MS: 283.05 [M−H]^−^; Anal. Calcd. For C_14_H_8_N_2_O_5_ (MW: 284.21 g/mol): C, 59.16%; H, 2.84%; N, 9.86%; Found: C, 59.15%; H, 2.83%; N, 9.88%.

*1.2.5. 2-(3-fluorophenyl)-7-nitro-4H-3,1-benzoxazin-4-one (ZAK-I-87)*

Yield: 79%; Yellow solid; mp: 104 °C; IR (υ_max_, KBr, cm^−1^): 1770, 1665; ^1^H NMR (CDCl_3_, 400 MHz) δ: 8.47 (d,1H, Ar–H, *J* = 2.2 Hz), 8.39 (d, 1H, Ar–H, *J* = 8.2), 8.24 (dd,1H, Ar–H, *J* = 2.4, 8.7 Hz), 7.76–7.68 (m, 3H, Ar–H) 7.28 (m,1H, Ar–H); ^13^C NMR (CDCl_3_, 75 MHz) δ: 164.3, 159.3, 154.4, 154.3, 152.6, 137.8, 132.2, 131.8, 130.2, 128.4, 124.2, 122.4, 119.6, 116.3; ESI-MS: 285.06 [M−H]−; Anal. Calcd. For C_14_H_7_FN_2_O_4_ (MW: 286.19 g/mol): C, 58.75%; H, 2.47%; N, 9.79%; Found: C, 58.71%; H, 2.50%; N, 9.80%.

*1.2.6. 2-(3-chlorophenyl)-7-nitro-4H-3,1-benzoxazin-4-one (ZAK-I-90)*

Yield: 75%; Brown solid; mp: 98 °C; IR (υ_max_, KBr, cm^−1^): 1758, 1628; ^1^H NMR (CDCl_3_, 400 MHz) δ: 8.53 (d, 1H, Ar–H, *J* = 2.1 Hz), 8.29 (dd,1H, Ar–H, *J* = 2.2, 8.6 Hz), 8.22–8.12 (m, 3H, Ar–H) 7.49–7.44 (m,2H, Ar–H); ^13^C NMR (CDCl_3_, 75 MHz) δ: 159.6, 156.8, 154.6, 152.2, 141.5, 137.6, 133.6, 132.6, 131.3, 129.8, 129.5, 128.6; ESI-MS: 301.46 [M−H]−; Anal. Calcd. For C_14_H_7_ClN_2_O_4_ (MW: 302.64 g/mol): C, 55.56%; H, 2.33%; N, 9.26%; Found: C, 55.53%; H, 2.35%; N, 9.27%.

*1.2.7. 2-[(E)-2-(4-fluorophenyl)ethenyl]-7-nitro-4H-3,1-benzoxazin-4-one (ZAK-I-93)*

Yield: 68%; Grey solid; mp: 138 °C; IR (υ_max_, KBr, cm^−1^): 1780, 1660, 978; 1H NMR (CDCl_3_, 400 MHz) δ: 8.55 (d,1H, Ar–H, *J* = 2.2 Hz), 8.37 (d, 1H, Ar–H, *J* = 8.1), 8.20 (dd,1H, Ar–H, *J* = 2.2, 8.2 Hz), 7.79 (d, 1H, CH=CH, *J* = 16.4 Hz), 7.63–7.32 (m, 4H, Ar–H), 6.76 (d, 1H, CH=CH, *J* = 16.2 Hz); ^13^C NMR (CDCl_3_, 75 MHz) δ:164.7, 159.5, 157.2, 156.4, 154.6, 147.1, 141.8, 136.4, 130.2, 128.1, 126.8, 119.2, 116.9, 116.4; ESI-MS: 311.04 [M−H]−; Anal. Calcd. For C_16_H_9_FN_2_O_4_ (MW: 312.21 g/mol): C, 61.54%; H, 2.91%; N, 8.97%; Found: C, 61.55%; H, 2.89%; N, 8.98%.

*1.2.8. 2-[(E)-2-(3,4-dimethoxyphenyl)ethenyl]-7-nitro-4H-3,1-benzoxazin-4-one (ZAK-I-97)*

Yield: 63%; Brown solid; mp: 152 °C; IR (υ_max_, KBr, cm^−1^):1764, 1651, 969; ^1^H NMR (CDCl_3_, 400 MHz) δ: 8.57 (d, 1H, Ar–H, *J* = 2.2 Hz), 8.36 (d, 1H, Ar–H, *J* = 8.0 Hz), 8.19 (dd, 1H, Ar–H, *J* = 2.2, 8.4 Hz), 7.74 (d, 1H, CH=CH, *J* = 16.3 Hz), 7.23–7.09 (m, 3H, Ar–H), 6.79 (d, 1H, CH=CH, *J* = 16.0 Hz), 3.90 (s, 6H, OCH_3_); ^13^C NMR (CDCl_3_, 75 MHz) δ: 158.6, 157.8, 156.4, 156.2, 152.4, 146.9, 136.0, 133.1, 132.5, 131.2, 128.3, 127.1, 120.4, 120.3, 116.6, 112.0, 56.1; ESI-MS: 353.18 [M−H]−; Anal. Calcd. For C_18_H_14_N_2_O_6_ (MW: 354.31 g/mol): C, 61.02%; H, 3.98%; N, 7.91%; Found: C, 61.01%; H, 3.98%; N, 7.92%.

1.3. DFT Calculations

The geometry of the chemotherapeutic drugs (ZAK-I-55, ZAK-I-57, ZAK-I-64, ZAK-I-87, ZAK-I-90, ZAK-I-93,) were optimized in this study utilizing DFT simulations conducted throughout the Gaussian 16 software with basis set B3LYP/6-31G.^1^ We selected this basis set because it is significantly related to the geometry optimization of such molecules. Relaxation of the structural features is the first step in our computation.

1.4. Computational systems pharmacology analysis

1.4.1 Physicochemical, pharmacokinetics, drug-likeness, and medicinal chemistry properties analysis

The physicochemical, pharmacokinetic, drug-likeness, and medicinal chemistry properties of benzoxazinone derivatives were proposed using the SwissADME online web tool.^2^

1.4.2 Prediction of potential targets of benzoxazinone derivatives

Potential protein targets of benzoxazinone derivatives (ZAK-I-55, ZAK-I-57, ZAK-I-64, ZAK-I-68, ZAK-I-87, ZAK-I-90, ZAK-I-93, and ZAK-I-97) were predicted using the SwissTargetPrediction online tool.^3^ After merging the datasets, 638 potential protein targets were identified with a probability score > 0. Since different compounds can share common targets, redundancy was removed by eliminating duplicates, ensuring each protein target was counted only once. This refinement resulted in 265 unique potential protein targets for further analysis.

1.4.3 HCC-related target screening

HCC-related targets were retrieved from two databases, OncoDB.HCC (http://oncodb.hcc.ibms.sinica.edu.tw) and Liverome (http://liverome.kobic.re.kr/index.php).^4–6^

1.4.4 Identification of intersection targets

Intersection targets were identified between the potential targets of benzoxazinone derivatives and HCC-related targets using the VENNY 2.1 online tool.^7^

1.4.5 Protein-protein interaction (PPI) analysis

PPI analysis was further carried out on identified intersected targets at a medium confidence score of 0.400 and species limited to “Homo sapiens” by employing the STRING database.^8^ The results of the STRING PPI analysis were further uploaded to the Cytoscape software (version 3.9.0, Boston, MA, USA) in a. tsv file format for further analysis to determine the potential anti-HCC core targets.^9^

1.4.6 Expression of anti-HCC core targets in Liver hepatocellular carcinoma (LHIC) analysis

The expression of the top six anti-HCC core targets in LHIC was analyzed using the GEPIA database.^10^

1.4.7 Network construction between anti-HCC targets and benzoxazinone derivatives

The network between anti-HCC targets and benzoxazinone derivatives was further constructed by employing Cytoscape software (version 3.9.0, Boston, MA, USA).^9^

1.4.8 GO and KEGG enrichment analysis

The GO and KEGG enrichment analysis were further performed on fifty intersected targets by employing the database for annotation, visualization, and integrated discovery (DAVID; Version 6.8) (https://david.ncifcrf.gov/).^11^ The GO terms were categorized into three types: cellular component (CC), biological process (BP), and molecular function (MF). By uploading the data to the Bioinformatics platform (http://www.bioinformatics.com.cn/), the top 10 GO analysis data (BP, CC, and MF) and top 30 KEGG pathways were further shown in the form of an enrichment dot bubble plot.^12^ The classical hypergeometric test was used to determine statistical significance. The adjusted p ≤ 0.05 was utilized as the significant threshold in our investigation.

1.4.9 Molecular docking

Two-dimensional (2D) structures of benzoxazinone derivatives were generated using ChemDraw Ultra (version 12.0) and saved in a MOL file (. mol) format. Three-dimensional (3D) structures were generated and saved in PDB format by uploading each MOL file (. mol) to BIOVIA Discovery Studio Visualizer software. The Protein Data Bank (https://www.rcsb.org/) was used to obtain the crystal structures of the anti-HCC core targets (c-Myc 5I4Z, ESR1 1R5K, EGFR 5Y9T, HSP90AA1 4BQG, CCND1 5VZU, and ERBB2 2A91).^13^ The ligands and water molecules from each targeted protein were hauled out, and subsequently, grid construction was also performed using the BIOVIA Discovery Studio Visualizer software.^14^ PDB files were uploaded to AutoDock Vina (version 1.2.0). and receptor proteins were charged with Kollman and Gasteiger partial charges. Benzoxazinone derivatives were uploaded to AutoDock Vina (version 1.2.0). in PDB format. AutoDock Vina (version 1.2.0.) was used to convert both proteins and benzoxazinone derivatives to the pdbqt format. Subsequently, proteins and benzoxazinone derivatives in pdbqt format were used to write scripts for molecular docking using AutoDock Vina (version 1.2.0.), and docked complex findings were acquired.^15^ The docked complexes were further analyzed to determine the binding capabilities of the molecules’ and targets’ through various interactions using the BIOVIA Discovery Studio Visualizer software.^14^ A binding energy < 0 implies that a ligand may instinctively bind to the receptor. It is commonly recognized that the lower the energy score of the ligand and receptor binding configuration, the more probable the binding will occur.^15^

**
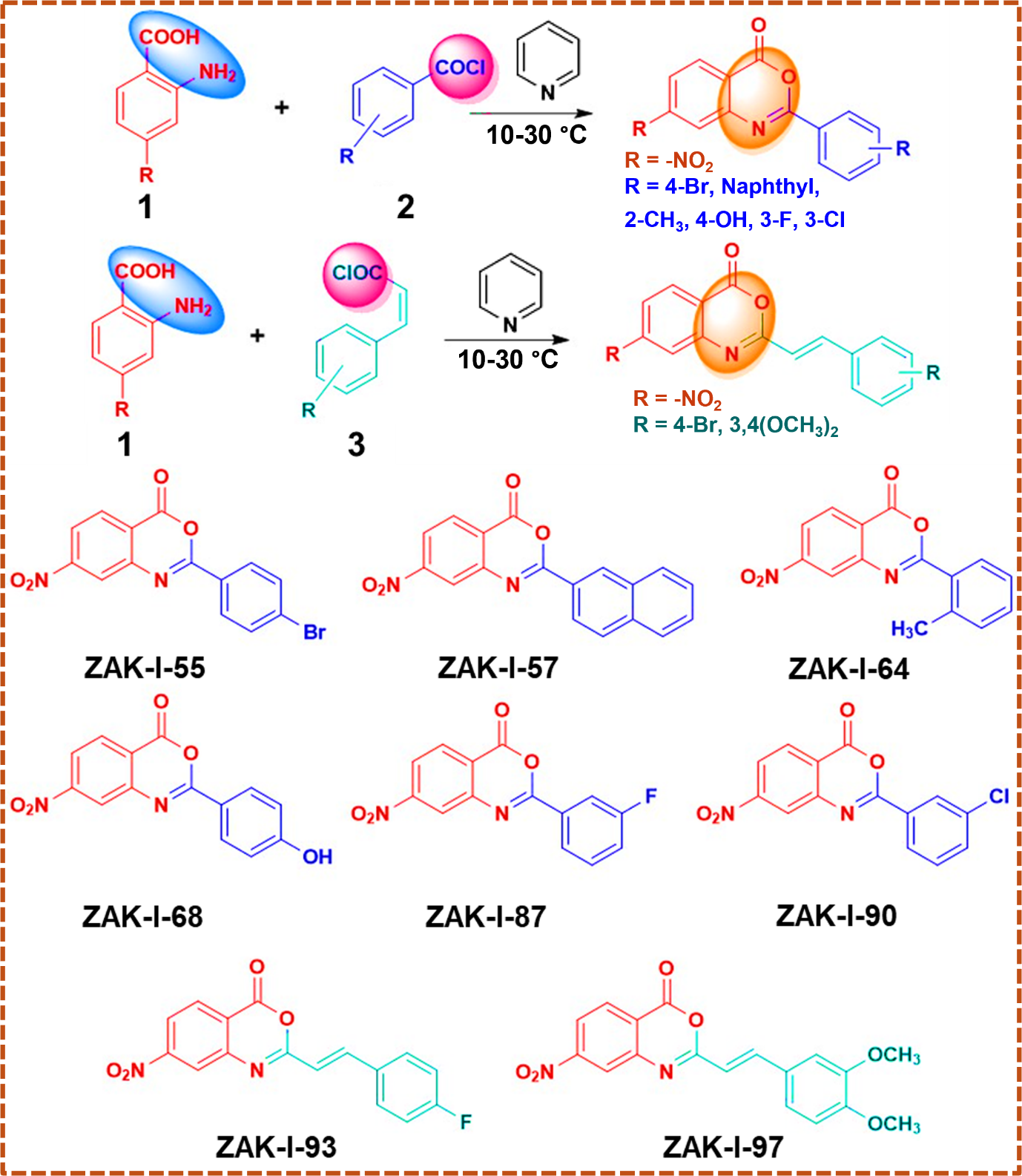
**

**Figure S1.** Scheme for the synthesis of benzoxazinone derivatives.

**
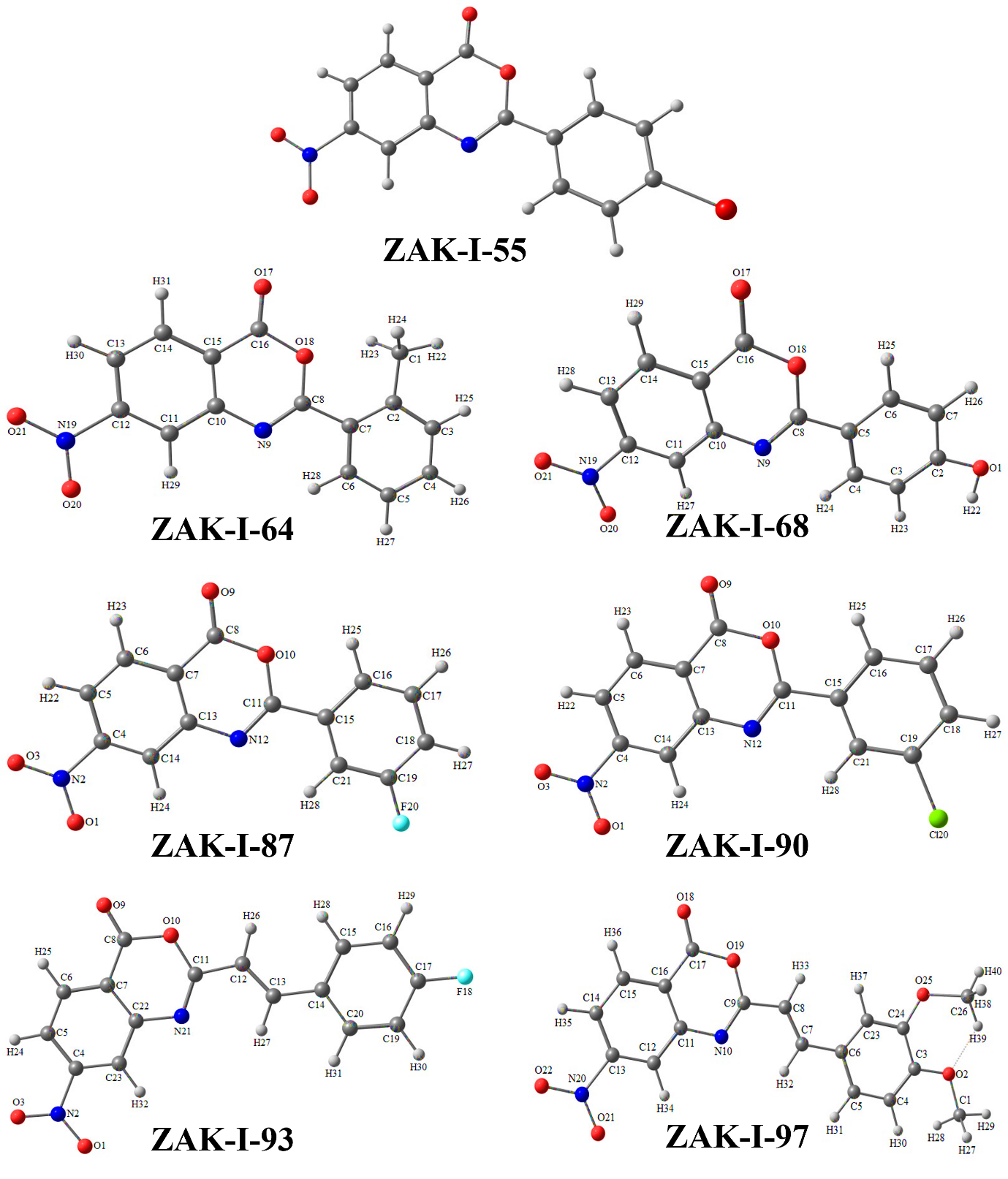
**

**Figure S2.** Optimized geometry diagrams of ZAK-I-55, ZAK-I-64, ZAK-I-68, ZAK-I-87, ZAK-I-90, ZAK-I-93, and ZAK-I-97.

**
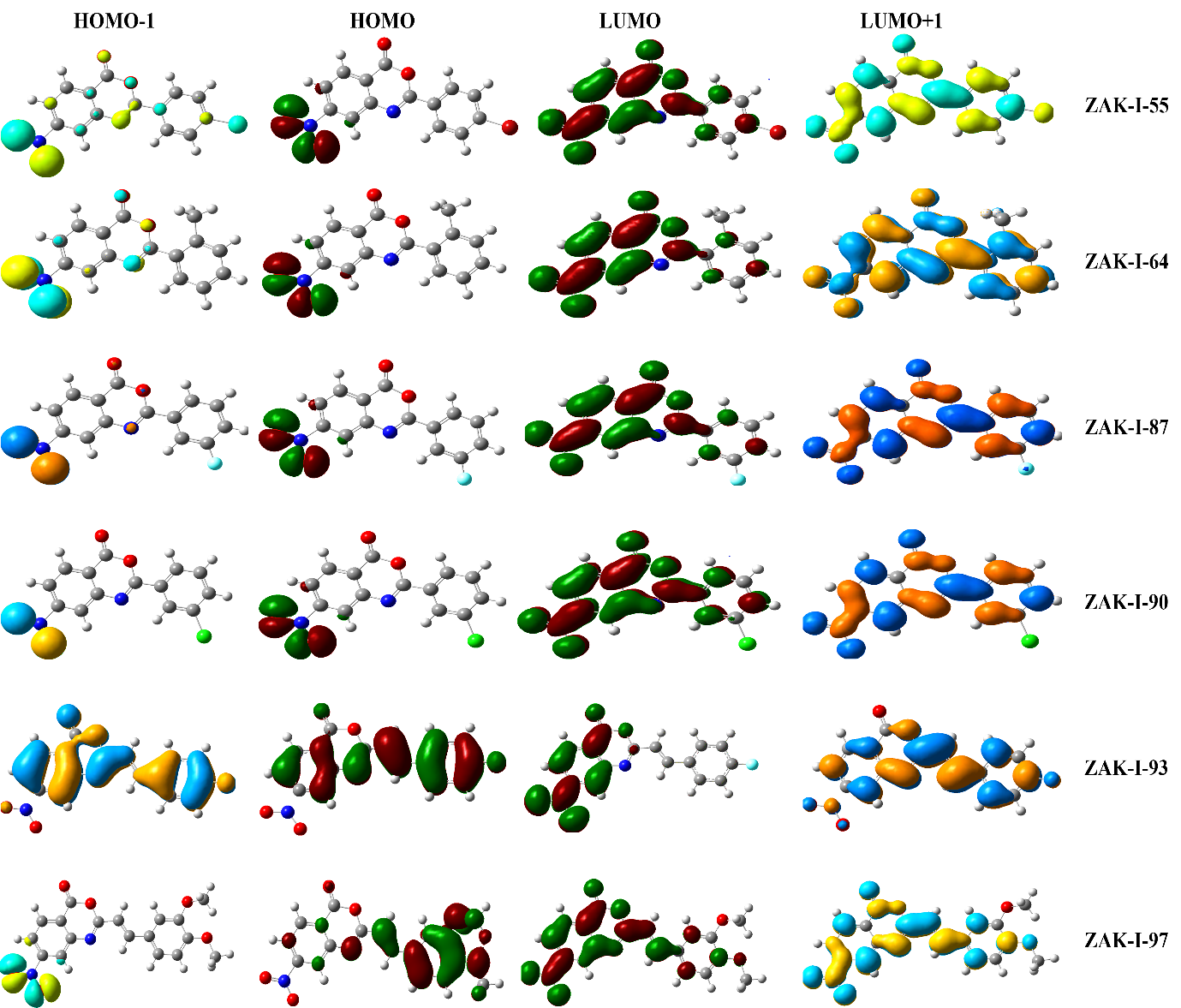
**

**Figure S3.** Frontier molecular orbitals of benzoxazinone derivatives. HOMO-1, HOMO, LUMO, and LUMO+1 orbitals of ZAK-I-55, ZAK-I-64, ZAK-I-68, ZAK-I-87, ZAK-I-90, ZAK-I-93, and ZAK-I-97.

**
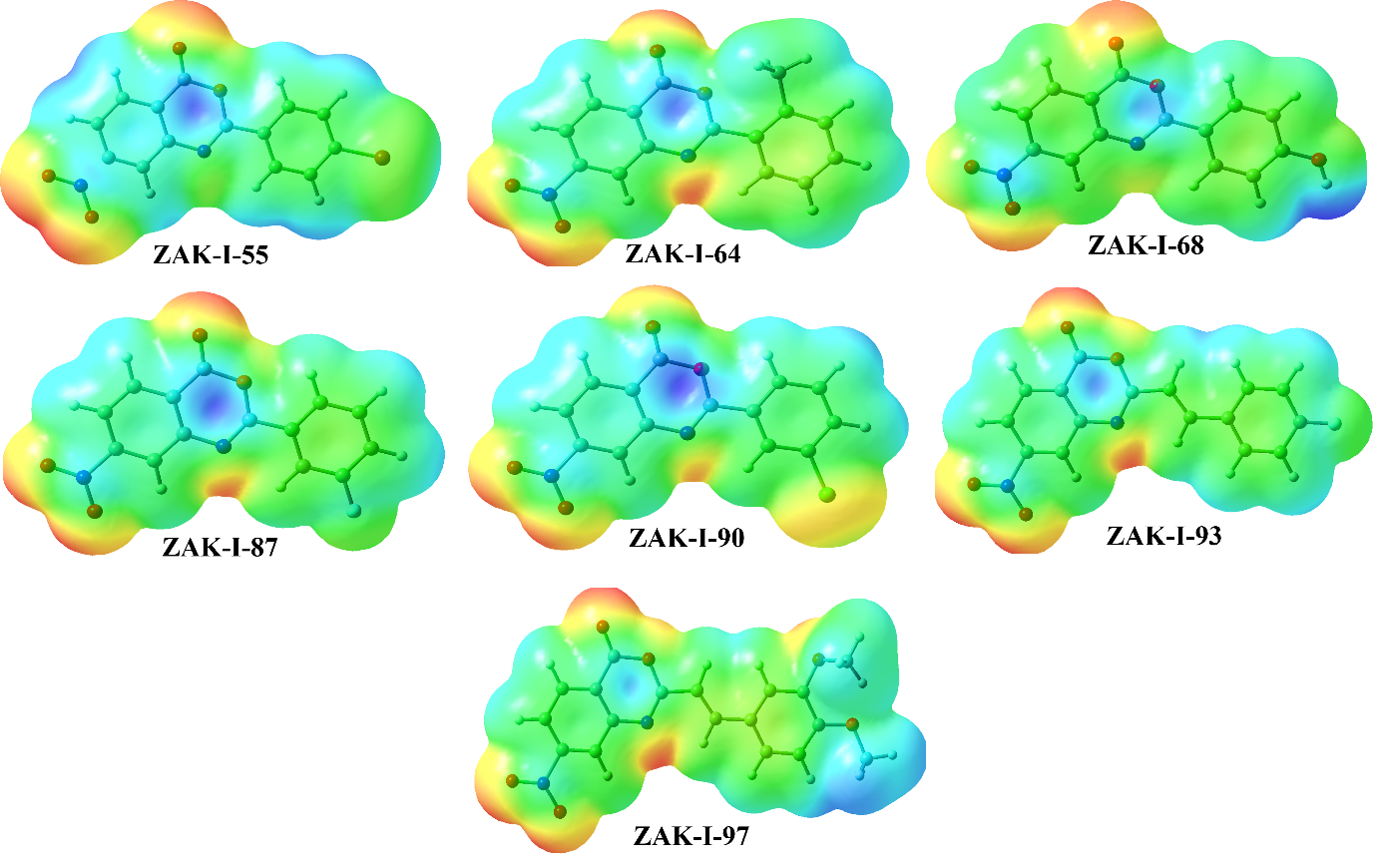
**

**Figure S4.** Molecular electrostatic potential (MEP) mapping of ZAK-I-55, ZAK-I-64, ZAK-I-68, ZAK-I-87, ZAK-I-90, ZAK-I-93, and ZAK-I-97. Green, orange, blue, red, and yellow colors on MEP surfaces indicate the order of magnitude of the electrostatic potential throughout the structures. The colors are arranged in the following order: red, orange, yellow, green, and blue.


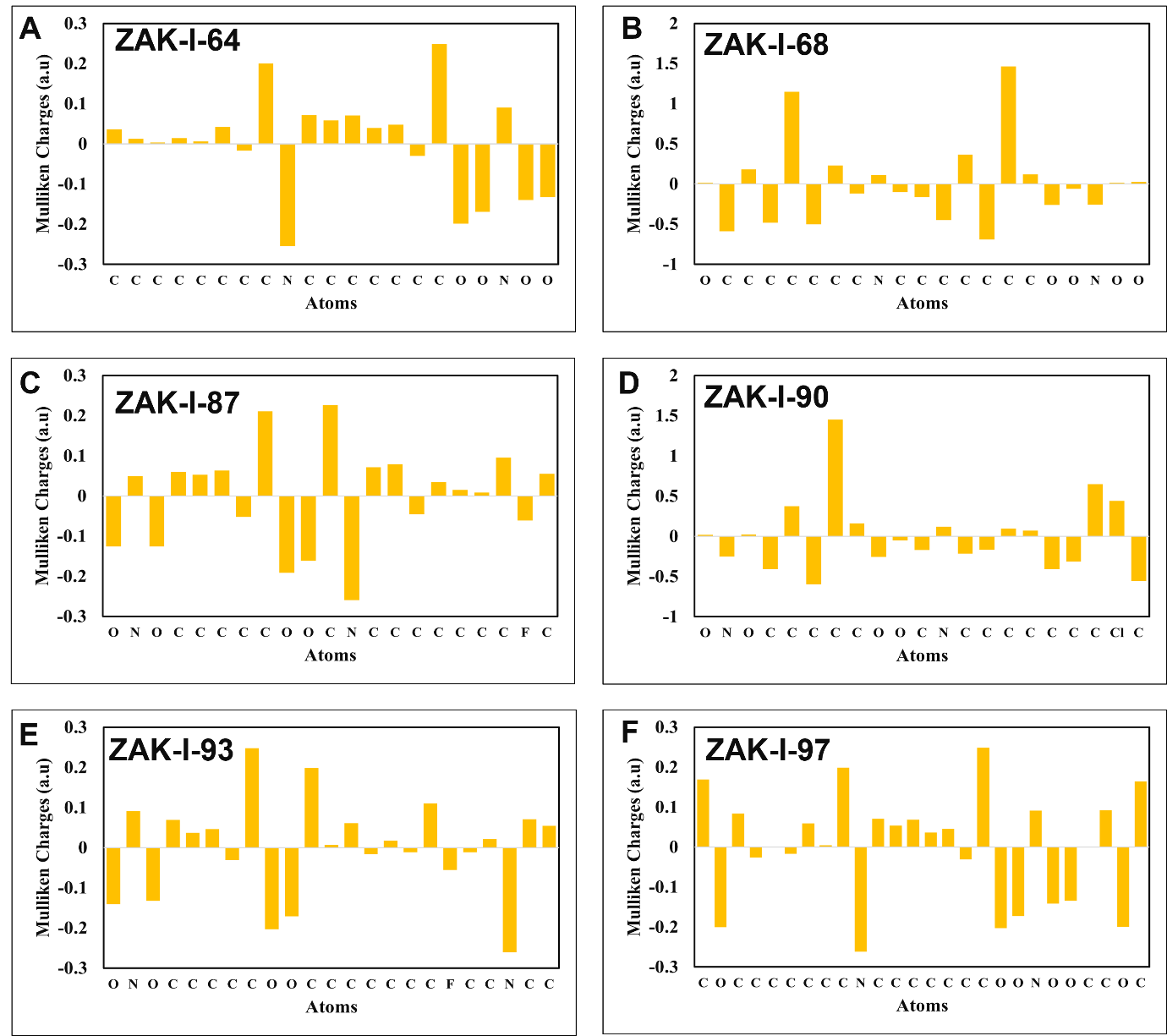


**Figure S5.** Mulliken charges spectra of ZAK-I-55, ZAK-I-64, ZAK-I-68, ZAK-I-87, ZAK-I-90, ZAK-I-93, and ZAK-I-97.


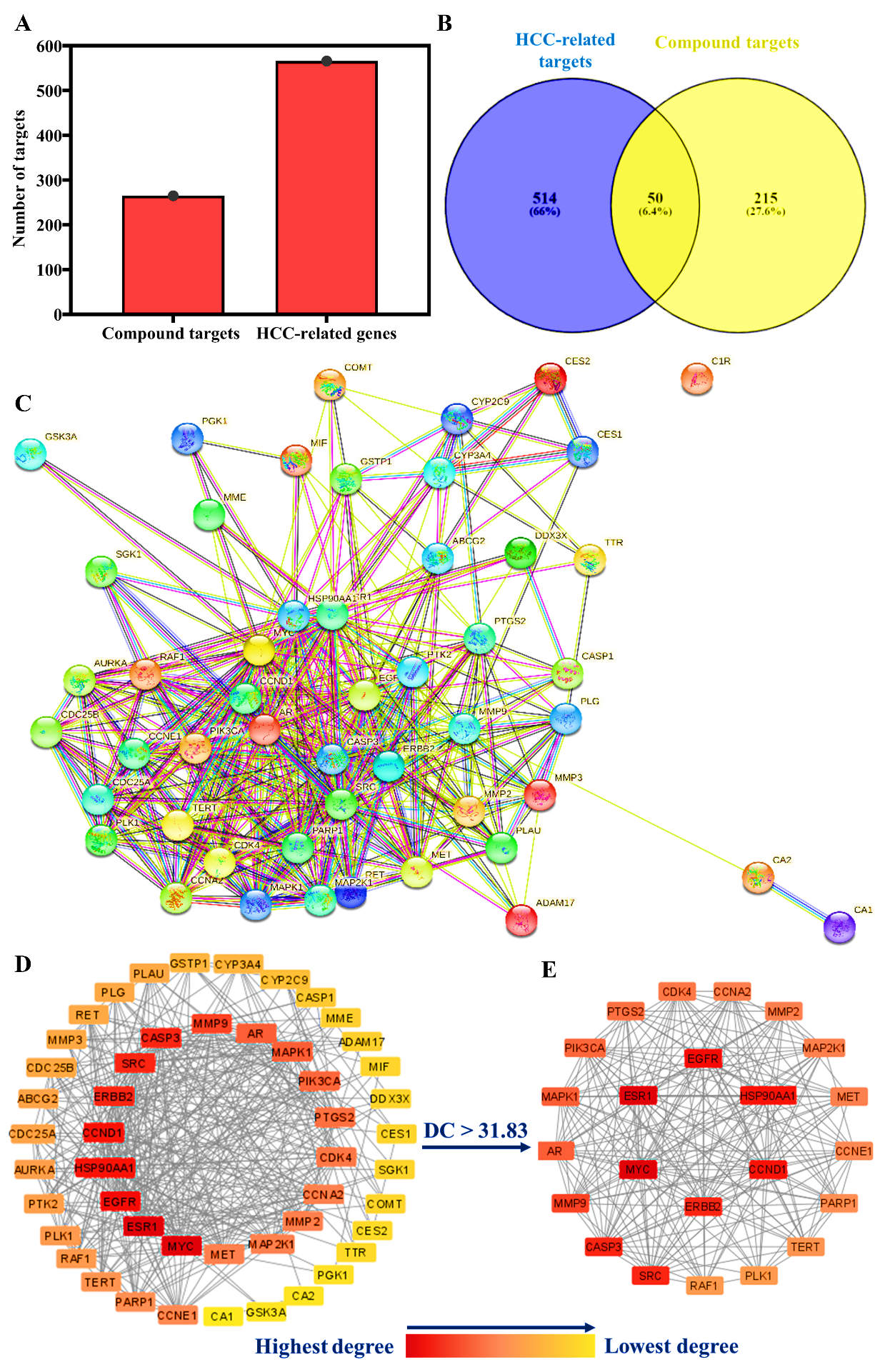


**Figure S6.** Identification of anti-HCC core targets via intersection and PPI network analysis**. (A)** Potential protein targets of benzoxazinone derivatives and HCC-related targets. **(B**) Intersecting targets between HCC-related targets and potential protein targets of benzoxazinone derivatives. **(C)** A PPI network of 50 intersecting targets was constructed using the STRING database. **(D)** PPI network of 50 intersecting targets and **(E)** 23 potential anti-HCC core targets were constructed using Cytoscape software. Each node is represented by a color ranging from red (highest) to yellow (lowest), as the degree of the node decreases. DC denotes degree of centrality.

**
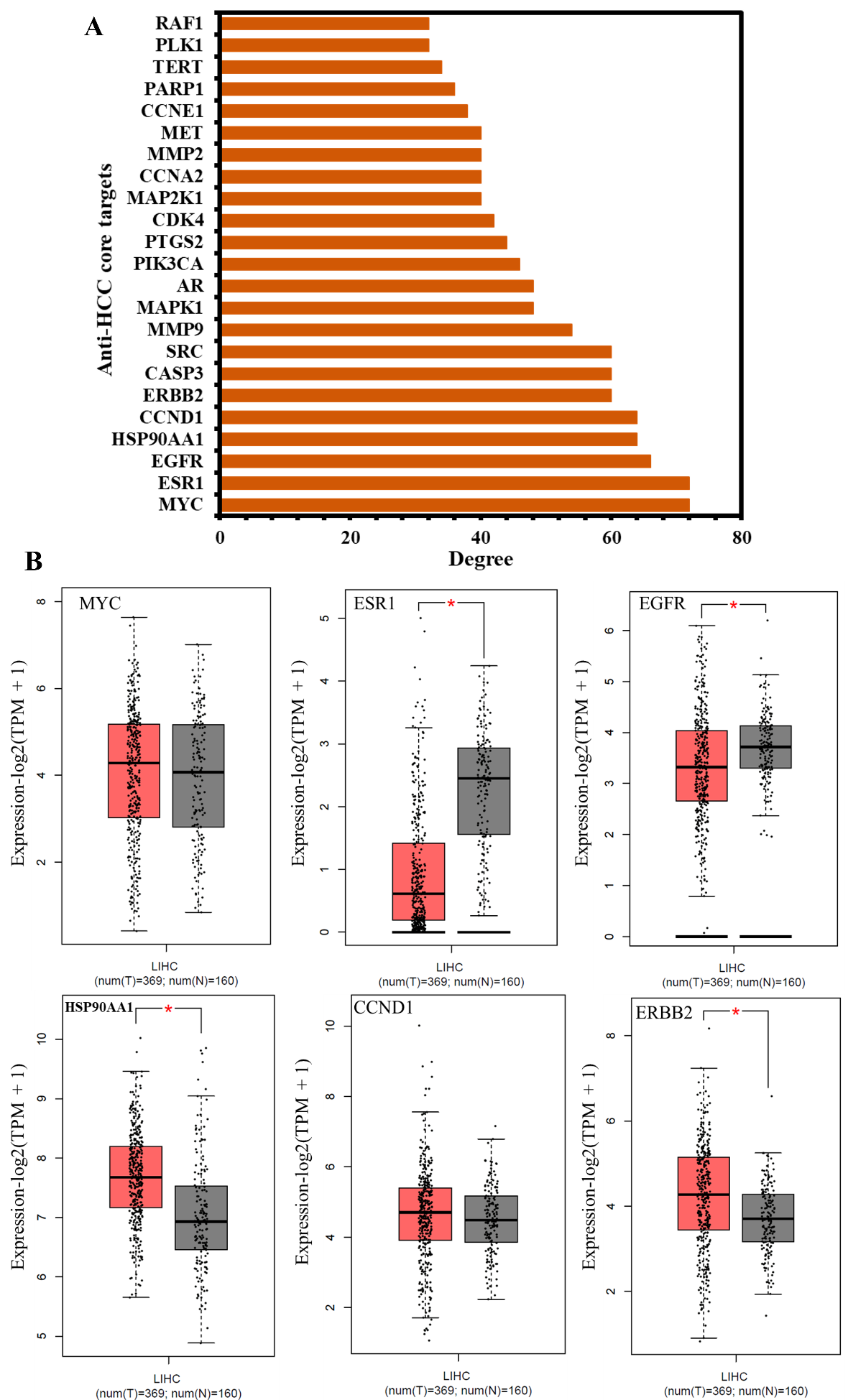
**

**Figure S7.** Anti-HCC core targets ranking and differential expression in LIHC**. (A)** The 23 anti-HCC core targets were ranked by a DC value greater than the average value (31.83). (**B**) Expression of top six anti-HCC core targets in the LHIC and normal samples.

**
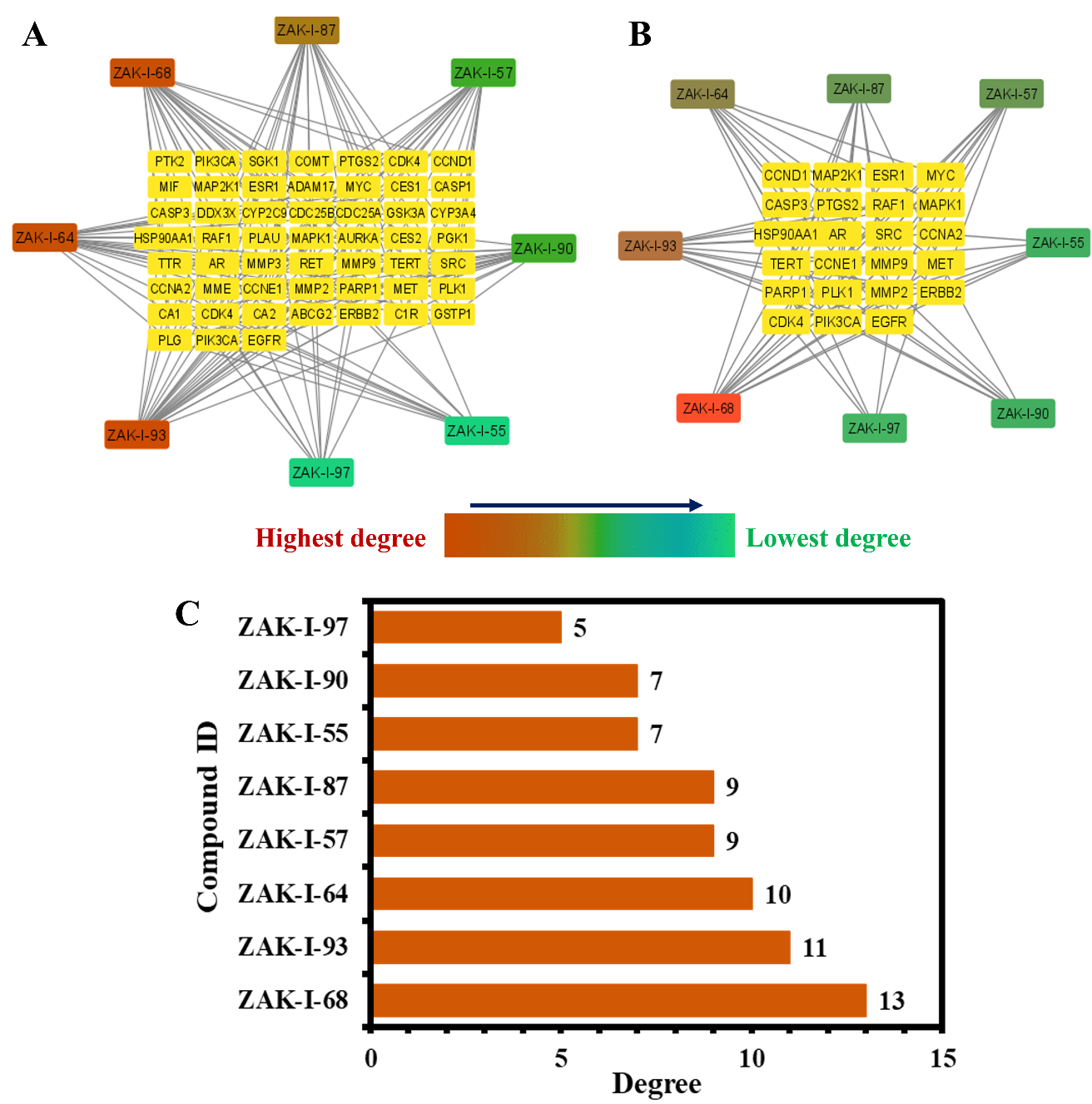
**

**Figure S8.** Compound–target and hub network analysis of benzoxazinone derivatives. (**A**) Network of eight benzoxazinone derivatives with 50 anti-HCC targets. (**B**) Hub network between eight benzoxazinone derivatives and 23 anti-HCC core targets. Each node is represented by a color ranging from red (highest) to green (lowest), as the degree of the node decreases. (**C**) Eight benzoxazinone derivatives with respect to their degree values in hub network.

**
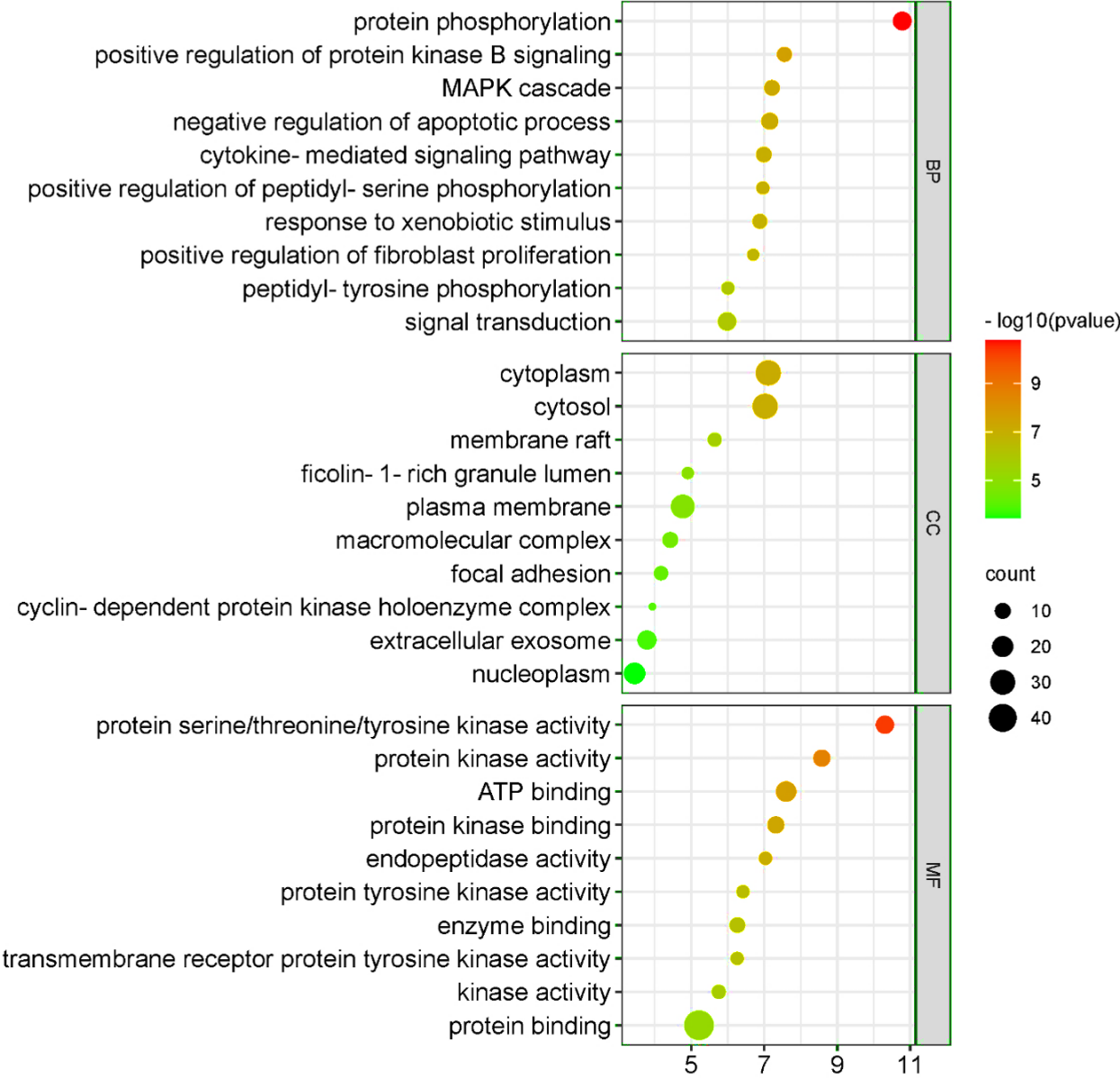
**

**Figure S9.** GO enrichment analysis of 50 anti-HCC targets implicated in HCC treatment with benzoxazinone derivatives.


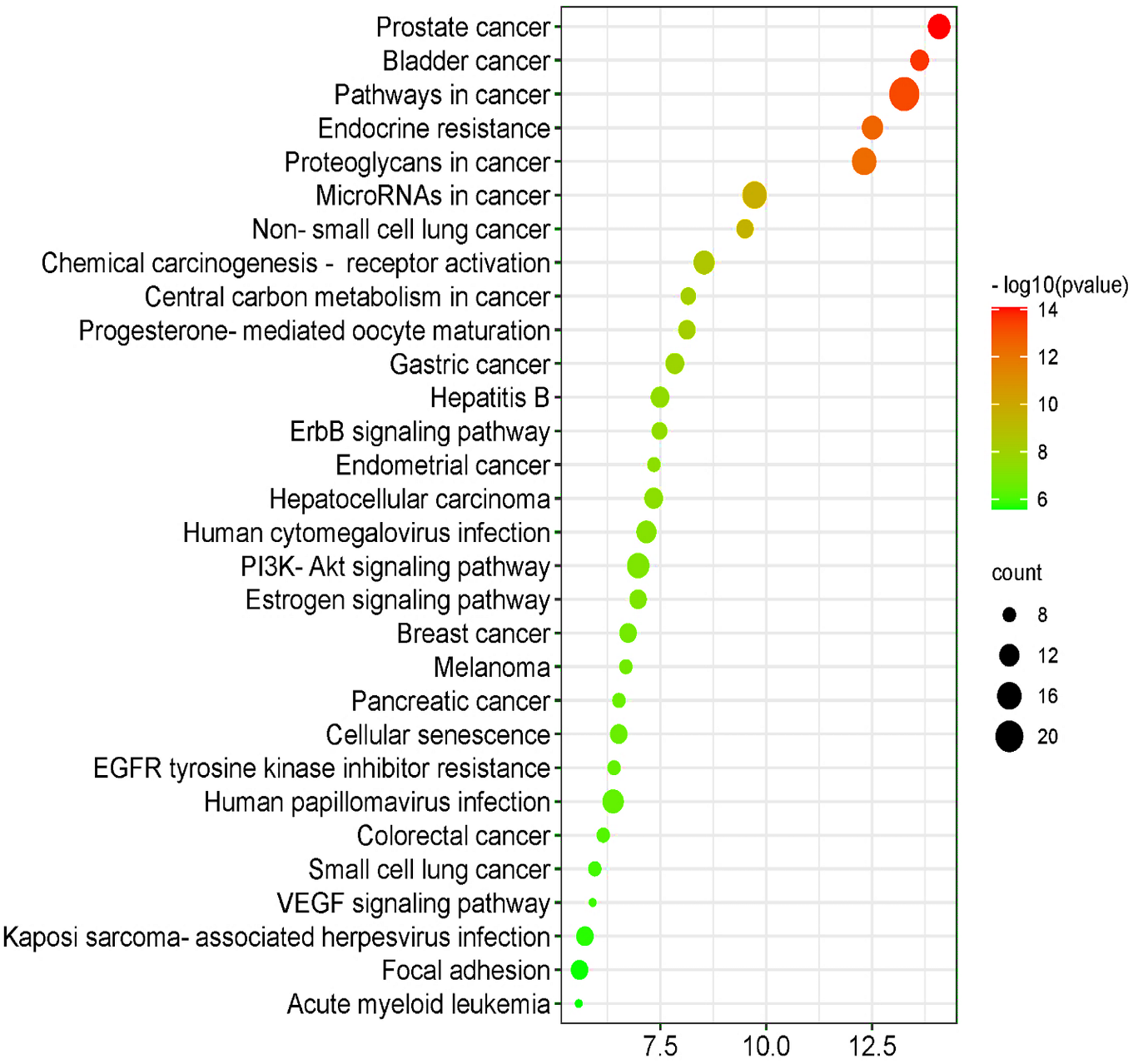


**Figure S10.** Top 30 KEGG signaling pathways involved in the anti-HCC therapeutic actions of benzoxazinone derivatives.


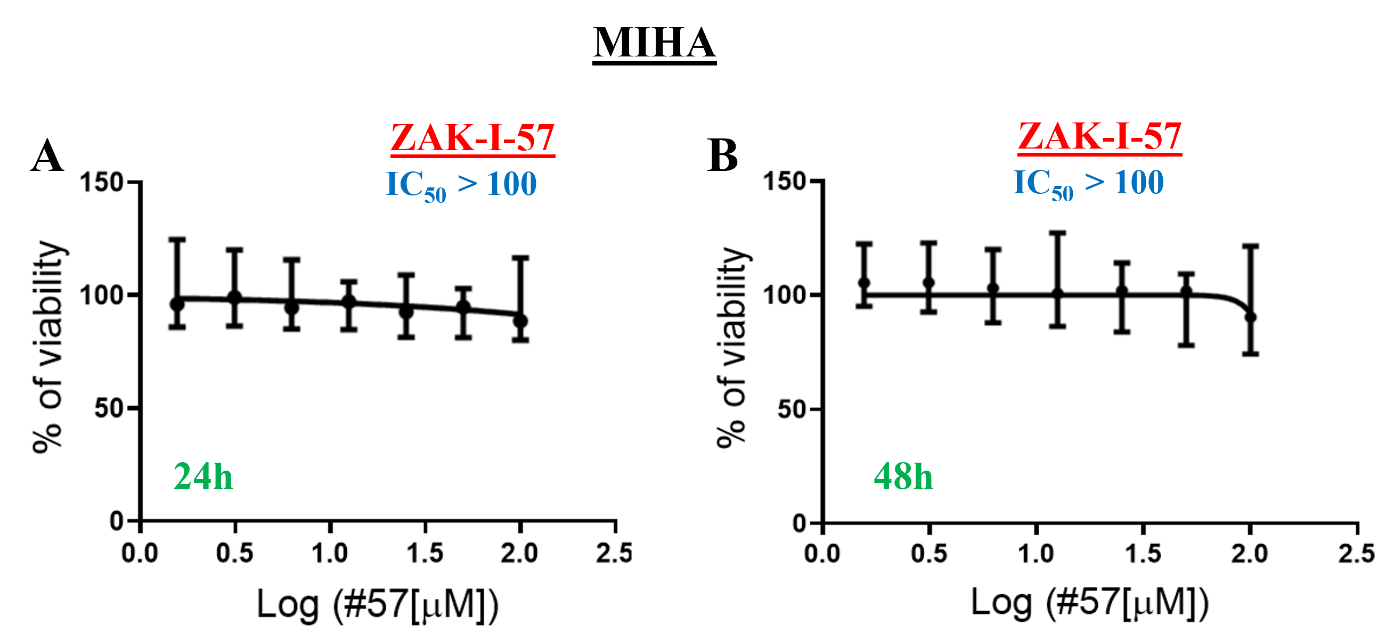


**Figure S11.** Cytotoxicity assessment of ZAK-I-57 in MIHA cells**.** The MTT results of (**A**) 24 h and (**B**) 48 h treatments with ZAK‐I‐57.


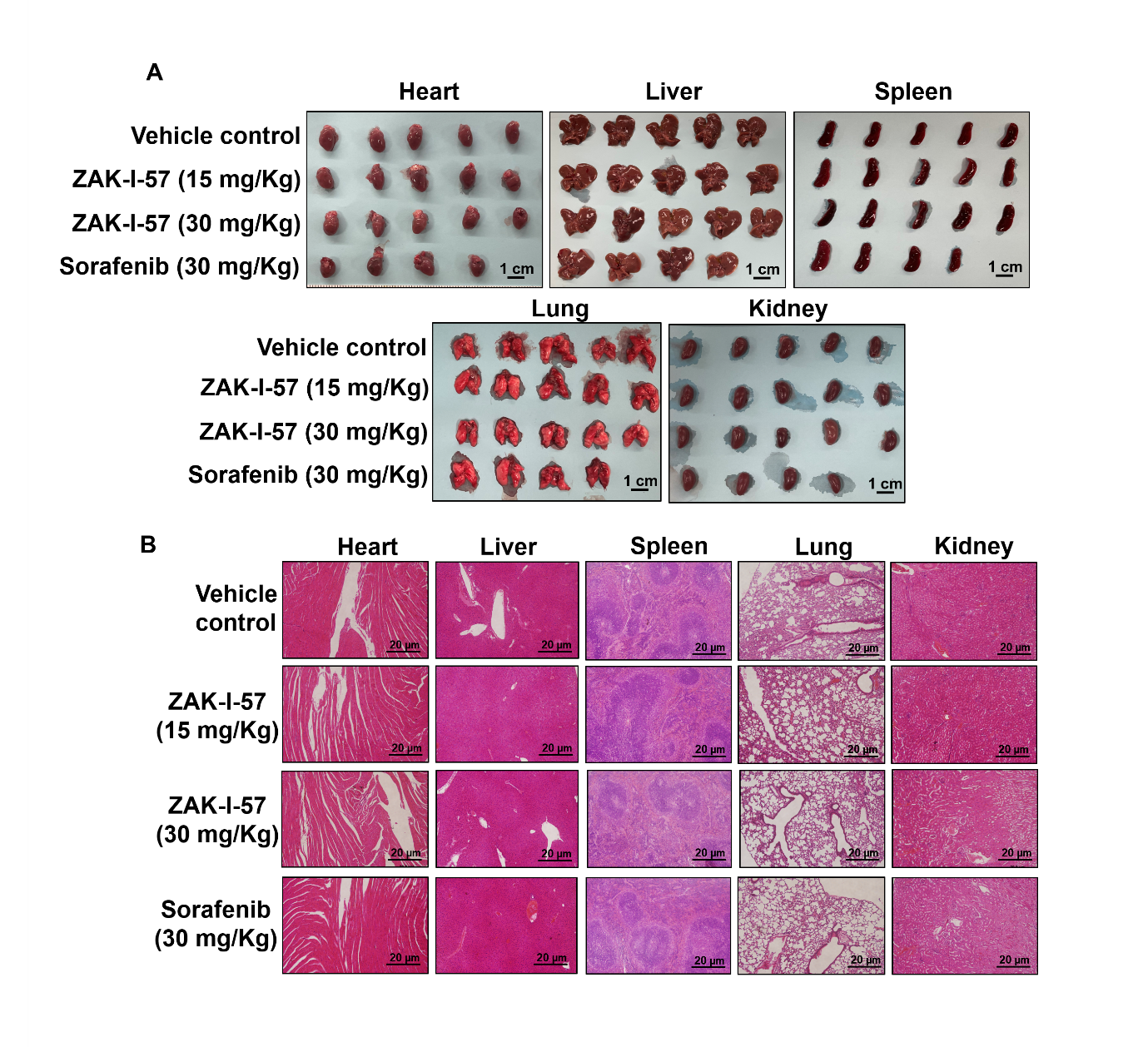


**Figure S12.** Effect of ZAK-I-57 on morphological and histological assessments of major organs in PDTX mouse model. (**A**) Gross anatomical assessment of vital organs (heart, liver, kidney, spleen, and lungs) from an HCC PDTX mouse model administered a control vehicle, sorafenib at a therapeutic dose of 30 mg/kg, and ZAK-I-57 at two doses (15 and 30 mg/kg). Scale bar=1 cm (**B**) Histopathological compendium delineates H&E-stained organ sections from an HCC PDTX mouse model administered a control vehicle, sorafenib at a therapeutic dose of 30 mg/kg, and ZAK-I-57 at two doses (15 and 30 mg/kg). Scale bar=20 µm.

**Table S1.** Reactivity indices for the benzoxazinone derivatives.

| Compounds | Electronegativity (χ) | Potential (μ) | Hardness (η) | Softness (S) | Electrophilicity index (ω) |  |
| --- | --- | --- | --- | --- | --- | --- |
|  |  |  |  |  |  |  |
| ZAK-I-55 | 5.54 | -5.54 | 1.49 | 0.34 | 10.32 |  |
| ZAK-I-57 | 2.25 | -2.25 | 1.99 | 0.25 | 1.27 |  |
| ZAK-I-64 | 2.24 | -2.24 | 2.00 | 0.25 | 1.25 |  |
| ZAK-I-68 | 4.32 | -4.32 | 2.70 | 0.19 | 3.46 |  |
| ZAK-I-87 | 2.28 | -2.28 | 1.99 | 0.25 | 1.31 |  |
| ZAK-I-90 | 5.74 | -5.74 | 1.60 | 0.31 | 10.30 |  |
| ZAK-I-93 | 5.22 | -5.22 | 1.28 | 0.39 | 10.64 |  |
| ZAK-I-97 | 5.02 | -5.02 | 1.19 | 0.42 | 10.59 |  |

**Table S2.** Optimized geometrical parameters of ZAK-I-55.

| **Bond** | **length (A)** | **Bond** | **Angle (°)** |
| --- | --- | --- | --- |
| R(1-2) | 1.32 | A(1-2-3) | 126.0 |
| R(2-3) | 1.32 | A(1-2-4) | 117.1 |
| R(2-4) | 1.56 | A(3-2-4) | 116.9 |
| R(4-5) | 1.42 | A(2-4-5) | 118.2 |
| R(4-14) | 1.40 | A(2-4-14) | 118.7 |
| R(5-6) | 1.40 | A(5-4-14) | 123.1 |
| R(5-25) | 1.10 | A(4-5-6) | 118.3 |
| R(6-7) | 1.42 | A(4-5-25) | 118.7 |
| R(6-26) | 1.10 | A(4-14-13) | 118.7 |
| R(7-8) | 1.51 | A(4-14-27) | 120.1 |
| R(7-13) | 1.43 | A(6-5-25) | 122.9 |
| R(8-9) | 1.25 | A(5-6-7) | 120.1 |
| R(8-10) | 1.47 | A(5-6-26) | 121.1 |
| R(10-11) | 1.41 | A(7-6-26) | 118.7 |
| R(11-12) | 1.34 | A(6-7-8) | 119.5 |
| R(11-15) | 1.51 | A(6-7-13) | 120.9 |
| R(12-13) | 1.45 | A(8-7-13) | 119.6 |
| R(13-14) | 1.43 | A(7-8-9) | 127.8 |
| R(14-27) | 1.10 | A(7-8-10) | 115.5 |
| R(15-16) | 1.44 | A(7-13-12) | 123.3 |
| R(15-24) | 1.40 | A(7-13-14) | 118.8 |
| R(16-17) | 1.38 | A(9-8-10) | 116.7 |
| R(16-28) | 1.10 | A(8-10-11) | 117.9 |
| R(17-18) | 1.44 | A(10-11-12) | 129.5 |
| R(17-29) | 1.10 | A(10-11-15) | 111.3 |
| R(18-19) | 1.44 | A(12-11-15) | 119.2 |
| R(18-23) | 1.45 | A(11-12-13) | 114.2 |
| R(19-20) | 1.39 | A(11-15-16) | 120.3 |

**Table S3.** Optimized geometrical parameters of ZAK-I-57.

| **Bond** | **length (A)** | **Bond** | **Angle (°)** |
| --- | --- | --- | --- |
| R(1-2) | 1.54 | A(2-1-22) | 109.6 |
| R(1-22) | 1.10 | A(2-1-23) | 111.2 |
| R(1-23) | 1.10 | A(2-1-24) | 111.2 |
| R(1-24) | 1.10 | A(1-2-3) | 118.1 |
| R(2-3) | 1.42 | A(1-2-7) | 124.0 |
| R(2-7) | 1.43 | A(22-1-23) | 108.6 |
| R(3-4) | 1.41 | A(22-1-24) | 108.7 |
| R(3-25) | 1.10 | A(23-1-24) | 107.5 |
| R(4-5) | 1.41 | A(3-2-7) | 117.9 |
| R(4-26) | 1.10 | A(2-3-4) | 122.0 |
| R(5-6) | 1.40 | A(2-3-25) | 118.6 |
| R(5-27) | 1.10 | A(2-7-6) | 119.6 |
| R(6-7) | 1.42 | A(2-7-8) | 124.7 |
| R(6-28) | 1.10 | A(4-3-25) | 119.4 |
| R(7-8) | 1.52 | A(3-4-5) | 119.8 |
| R(8-9) | 1.35 | A(3-4-26) | 119.9 |
| R(8-18) | 1.41 | A(5-4-26) | 120.3 |
| R(9-10) | 1.45 | A(4-5-6) | 119.3 |
| R(10-11) | 1.43 | A(4-5-27) | 120.5 |
| R(10-15) | 1.43 | A(6-5-27) | 120.2 |
| R(11-12) | 1.40 | A(5-6-7) | 121.4 |
| R(11-29) | 1.10 | A(5-6-28) | 121.4 |
| R(12-13) | 1.42 | A(7-6-28) | 117.2 |
| R(12-19) | 1.56 | A(6-7-8) | 115.7 |
| R(13-14) | 1.40 | A(7-8-9) | 117.9 |
| R(13-30) | 1.10 | A(7-8-18) | 113.4 |
| R(14-15) | 1.42 | A(9-8-18) | 128.7 |
| R(14-31) | 1.10 | A(8-9-10) | 114.7 |

**Table S4.** Optimized geometrical parameters of ZAK-I-64.

| **Bond** | **length (A)** | **Bond** | | **Angle (°)** | |
| --- | --- | --- | --- | --- | --- |
| R(1-2) | 1.31 | | A(3-2-4) | | 116.9 |
| R(4-5) | 1.42 | | A(2-4-5) | | 118.2 |
| R(4-23) | 1.40 | | A(2-4-23) | | 118.6 |
| R(15-36) | 1.10 | | A(7-8-33) | | 122.6 |
| R(16-17) | 1.51 | | A(9-8-33) | | 115.3 |
| R(17-18) | 1.25 | | A(8-9-10) | | 120.4 |
| R(17-19) | 1.47 | | A(8-9-19) | | 110.1 |
| R(20-21) | 1.32 | | A(10-9-19) | | 129.5 |
| R(20-22) | 1.32 | | A(9-10-11) | | 114.1 |
| R(23-24) | 1.41 | | A(9-19-17) | | 117.8 |
| R(23-37) | 1.10 | | A(10-11-12) | | 117.9 |
| R(24-25) | 1.43 | | A(10-11-16) | | 123.4 |
| R(25-26) | 1.48 | | A(12-11-16) | | 118.6 |
| R(26-38) | 1.11 | | A(11-12-13) | | 118.7 |
| R(26-39) | 1.11 | | A(11-12-34) | | 121.2 |
| R(26-40) | 1.11 | | A(11-16-15) | | 120.9 |
| R(2-39) | 1.92 | | A(11-16-17) | | 119.6 |
|  |  | | A(13-12-34) | | 120.1 |
|  |  | | A(12-13-14) | | 123.2 |
|  |  | | A(12-13-20) | | 118.6 |
|  |  | | A(14-13-20) | | 118.2 |
|  |  | | A(13-14-15) | | 118.3 |
|  |  | | A(13-14-35) | | 118.8 |
|  |  | | A(13-20-21) | | 117.1 |
|  |  | | A(13-20-22) | | 116.9 |
|  |  | | A(15-14-35) | | 122.9 |
|  |  | | A(14-15-16) | | 120.2 |
|  |  | | A(14-15-36) | | 121.1 |
|  |  | | A(16-15-36) | | 118.7 |

**Table S5.** Optimized geometrical parameters of ZAK-I-68.

| **Bond** | **length (A)** | **Bond** | **Angle (°)** |
| --- | --- | --- | --- |
| R(1-2) | 1.41 | A(2-1-22) | 103.4 |
| R(1-22) | 1.03 | A(1-2-3) | 123.3 |
| R(2-3) | 1.43 | A(1-2-7) | 117.0 |
| R(2-7) | 1.43 | A(3-2-7) | 119.8 |
| R(3-4) | 1.40 | A(2-3-4) | 119.8 |
| R(3-23) | 1.10 | A(2-3-23) | 119.7 |
| R(4-5) | 1.42 | A(2-7-6) | 119.8 |
| R(4-24) | 1.10 | A(2-7-26) | 119.0 |
| R(5-6) | 1.42 | A(4-3-23) | 120.5 |
| R(5-8) | 1.50 | A(3-4-5) | 120.7 |
| R(6-7) | 1.40 | A(3-4-24) | 121.0 |
| R(6-25) | 1.10 | A(5-4-24) | 118.4 |
| R(7-26) | 1.10 | A(4-5-6) | 119.3 |
| R(8-9) | 1.35 | A(4-5-8) | 119.6 |
| R(8-18) | 1.42 | A(6-5-8) | 121.1 |
| R(9-10) | 1.45 | A(5-6-7) | 120.7 |
| R(10-11) | 1.43 | A(5-6-25) | 118.9 |
| R(10-15) | 1.43 | A(5-8-9) | 119.2 |
| R(11-12) | 1.40 | A(5-8-18) | 111.3 |
| R(11-27) | 1.10 | A(7-6-25) | 120.4 |
| R(12-13) | 1.42 | A(6-7-26) | 121.2 |
| R(12-19) | 1.56 | A(9-8-18) | 129.4 |
| R(13-14) | 1.40 | A(8-9-10) | 114.2 |
| R(13-28) | 1.10 | A(8-18-16) | 117.9 |
| R(14-15) | 1.42 | A(9-10-11) | 117.9 |
| R(14-29) | 1.10 | A(9-10-15) | 123.4 |
| R(15-16) | 1.51 | A(11-10-15) | 118.7 |
| R(16-17) | 1.25 | A(10-11-12) | 118.7 |

**Table S6.** Optimized geometrical parameters of ZAK-I-87.

| **Bond** | | **length (A)** | **Bond** | | **Angle (°)** | |
| --- | --- | --- | --- | --- | --- | --- |
| R(1-2) | 1.322 | | | A(1-2-3) | | 126.0 |
| R(2-3) | 1.322 | | | A(1-2-4) | | 117.1 |
| R(2-4) | 1.560 | | | A(3-2-4) | | 116.9 |
| R(4-5) | 1.416 | | | A(2-4-5) | | 118.3 |
| R(4-14) | 1.399 | | | A(2-4-14) | | 118.6 |
| R(5-6) | 1.402 | | | A(5-4-14) | | 123.1 |
| R(5-22) | 1.099 | | | A(4-5-6) | | 118.4 |
| R(6-7) | 1.420 | | | A(4-5-22) | | 118.7 |
| R(6-23) | 1.100 | | | A(4-14-13) | | 118.7 |
| R(7-8) | 1.510 | | | A(4-14-24) | | 120.1 |
| R(7-13) | 1.429 | | | A(6-5-22) | | 122.9 |
| R(8-9) | 1.249 | | | A(5-6-7) | | 120.1 |
| R(8-10) | 1.472 | | | A(5-6-23) | | 121.1 |
| R(10-11) | 1.414 | | | A(7-6-23) | | 118.7 |
| R(11-12) | 1.344 | | | A(6-7-8) | | 119.5 |
| R(11-15) | 1.514 | | | A(6-7-13) | | 120.9 |
| R(12-13) | 1.449 | | | A(8-7-13) | | 119.6 |
| R(13-14) | 1.427 | | | A(7-8-9) | | 127.8 |
| R(14-24) | 1.100 | | | A(7-8-10) | | 115.5 |
| R(15-16) | 1.418 | | | A(7-13-12) | | 123.2 |
| R(15-21) | 1.415 | | | A(7-13-14) | | 118.8 |
| R(16-17) | 1.408 | | | A(9-8-10) | | 116.7 |
| R(16-25) | 1.097 | | | A(8-10-11) | | 117.9 |
| R(17-18) | 1.406 | | | A(10-11-12) | | 129.6 |
| R(17-26) | 1.099 | | | A(10-11-15) | | 111.3 |
| R(18-19) | 1.426 | | | A(12-11-15) | | 119.1 |
| R(18-27) | 1.098 | | | A(11-12-13) | | 114.2 |
| R(19-20) | 1.370 | | | A(11-15-16) | | 120.7 |

**Table S7.** Optimized geometrical parameters of ZAK-I-90.

| **Bond** | **length (A)** | **Bond** | **Angle (°)** |
| --- | --- | --- | --- |
| R(1-2) | 1.32 | A(1-2-3) | 126.1 |
| R(2-3) | 1.32 | A(1-2-4) | 117.1 |
| R(2-4) | 1.56 | A(3-2-4) | 116.8 |
| R(4-5) | 1.42 | A(2-4-5) | 118.2 |
| R(4-14) | 1.40 | A(2-4-14) | 118.7 |
| R(5-6) | 1.40 | A(5-4-14) | 123.1 |
| R(5-22) | 1.10 | A(4-5-6) | 118.4 |
| R(6-7) | 1.42 | A(4-5-22) | 118.7 |
| R(6-23) | 1.10 | A(4-14-13) | 118.6 |
| R(7-8) | 1.51 | A(4-14-24) | 120.2 |
| R(7-13) | 1.43 | A(6-5-22) | 122.9 |
| R(8-9) | 1.25 | A(5-6-7) | 120.1 |
| R(8-10) | 1.47 | A(5-6-23) | 121.1 |
| R(10-11) | 1.41 | A(7-6-23) | 118.8 |
| R(11-12) | 1.34 | A(6-7-8) | 119.5 |
| R(11-15) | 1.51 | A(6-7-13) | 120.9 |
| R(12-13) | 1.45 | A(8-7-13) | 119.7 |
| R(13-14) | 1.43 | A(7-8-9) | 127.9 |
| R(14-24) | 1.10 | A(7-8-10) | 115.4 |
| R(15-16) | 1.42 | A(7-13-12) | 123.2 |
| R(15-21) | 1.42 | A(7-13-14) | 118.9 |
| R(16-17) | 1.41 | A(9-8-10) | 116.6 |
| R(16-25) | 1.10 | A(8-10-11) | 117.7 |
| R(17-18) | 1.41 | A(10-11-12) | 129.9 |
| R(17-26) | 1.10 | A(10-11-15) | 111.2 |
| R(18-19) | 1.41 | A(12-11-15) | 118.9 |
| R(18-27) | 1.10 | A(11-12-13) | 114.1 |
| R(19-20) | 1.81 | A(11-15-16) | 121.0 |

**Table S8.** Optimized geometrical parameters of ZAK-I-93.

| **Bond** | **length (A)** | **Bond** | **Angle (°)** |
| --- | --- | --- | --- |
| R(1-2) | 1.32 | A(1-2-3) | 126.0 |
| R(2-3) | 1.32 | A(1-2-4) | 117.1 |
| R(2-4) | 1.56 | A(3-2-4) | 116.9 |
| R(4-5) | 1.42 | A(2-4-5) | 118.2 |
| R(4-23) | 1.40 | A(2-4-23) | 118.6 |
| R(5-6) | 1.40 | A(5-4-23) | 123.1 |
| R(5-24) | 1.10 | A(4-5-6) | 118.3 |
| R(6-7) | 1.42 | A(4-5-24) | 118.8 |
| R(6-25) | 1.10 | A(4-23-22) | 118.7 |
| R(7-8) | 1.51 | A(4-23-32) | 120.1 |
| R(7-22) | 1.43 | A(6-5-24) | 122.9 |
| R(8-9) | 1.25 | A(5-6-7) | 120.2 |
| R(8-10) | 1.47 | A(5-6-25) | 121.1 |
| R(10-11) | 1.42 | A(7-6-25) | 118.7 |
| R(11-12) | 1.49 | A(6-7-8) | 119.5 |
| R(11-21) | 1.35 | A(6-7-22) | 120.9 |
| R(12-13) | 1.36 | A(8-7-22) | 119.6 |
| R(12-26) | 1.10 | A(7-8-9) | 127.7 |
| R(13-14) | 1.48 | A(7-8-10) | 115.5 |
| R(13-27) | 1.10 | A(7-22-21) | 123.4 |
| R(14-15) | 1.43 | A(7-22-23) | 118.7 |
| R(14-20) | 1.43 | A(9-8-10) | 116.8 |
| R(15-16) | 1.40 | A(8-10-11) | 117.7 |
| R(15-28) | 1.10 | A(10-11-12) | 110.1 |
| R(16-17) | 1.43 | A(10-11-21) | 129.6 |
| R(16-29) | 1.10 | A(12-11-21) | 120.3 |
| R(17-18) | 1.37 | A(11-12-13) | 122.0 |
| R(17-19) | 1.42 | A(11-12-26) | 115.3 |

**Table S9.** Optimized geometrical parameters of ZAK-I-97.

| **Bond** | **length (A)** | **Bond** | | **Angle (°)** | |
| --- | --- | --- | --- | --- | --- |
| R(1-2) | 1.48 | | A(2-1-27) | | 112.2 |
| R(1-27) | 1.11 | | A(2-1-28) | | 111.9 |
| R(1-28) | 1.11 | | A(2-1-29) | | 105.2 |
| R(1-29) | 1.11 | | A(1-2-3) | | 113.6 |
| R(2-3) | 1.42 | | A(1-2-39) | | 136.2 |
| R(3-4) | 1.42 | | A(27-1-28) | | 109.2 |
| R(3-24) | 1.45 | | A(27-1-29) | | 109.2 |
| R(4-5) | 1.40 | | A(28-1-29) | | 109.0 |
| R(4-30) | 1.09 | | A(2-3-4) | | 124.9 |
| R(5-6) | 1.42 | | A(2-3-24) | | 116.4 |
| R(5-31) | 1.10 | | A(3-2-39) | | 102.5 |
| R(6-7) | 1.48 | | A(4-3-24) | | 118.7 |
| R(6-23) | 1.42 | | A(3-4-5) | | 121.0 |
| R(7-8) | 1.36 | | A(3-4-30) | | 119.9 |
| R(7-32) | 1.10 | | A(3-24-23) | | 118.8 |
| R(8-9) | 1.49 | | A(3-24-25) | | 126.1 |
| R(8-33) | 1.10 | | A(5-4-30) | | 119.0 |
| R(9-10) | 1.35 | | A(4-5-6) | | 121.2 |
| R(9-19) | 1.42 | | A(4-5-31) | | 119.3 |
| R(10-11) | 1.44 | | A(6-5-31) | | 119.4 |
| R(11-12) | 1.43 | | A(5-6-7) | | 119.1 |
| R(11-16) | 1.43 | | A(5-6-23) | | 118.0 |
| R(12-13) | 1.40 | | A(7-6-23) | | 122.9 |
| R(12-34) | 1.10 | | A(6-7-8) | | 126.9 |
| R(13-14) | 1.42 | | A(6-7-32) | | 116.1 |
| R(13-20) | 1.56 | | A(6-23-24) | | 122.3 |
| R(14-15) | 1.40 | | A(6-23-37) | | 120.6 |
| R(14-35) | 1.10 | | A(8-7-32) | | 117.0 |

**Table S10.** Molecular orbital energies and other properties of the benzoxazinone derivatives.

| Compounds | E_HOMO_  (eV) | E_LUMO_  (eV) | ΔE=E_HOMO_–E_LUMO_  (eV) | Ionization Potential  *I* (eV) | Electron Affinity *A*  (eV) |
| --- | --- | --- | --- | --- | --- |
| ZAK-I-55 | -7.02 | -4.05 | 2.97 | 7.02 | 4.05 |
| ZAK-I-57 | -4.24 | -1.98 | 2.26 | 4.24 | 1.98 |
| ZAK-I-64 | -4.24 | -0.24 | 4.00 | 4.24 | 0.24 |
| ZAK-I-68 | -7.02 | -1.62 | 5.40 | 7.02 | 1.62 |
| ZAK-I-87 | -4.27 | -0.29 | 3.98 | 4.27 | 0.29 |
| ZAK-I-90 | -7.34 | -4.14 | 3.20 | 7.34 | 4.14 |
| ZAK-I-93 | -6.50 | -3.94 | 2.56 | 6.50 | 3.94 |
| ZAK-I-97 | -6.21 | -3.83 | 2.38 | 6.21 | 3.83 |

**Table S11**. Second-order pertbances theory analysis of NBO charges of ZAK-I-55.

| **Donor (i)** | **Type** | **Acceptor (j)** | **Type** | **E(2) Kcal/mol** | **E(j)-E(i) a.u** | **F(i.j) a.u** |
| --- | --- | --- | --- | --- | --- | --- |
| O1-N2 | π | N2-O3 | π^*^ | 6.16 | 1.07 | 0.073 |
| O1-N2 | π | C4-C5 | π^*^ | 0.66 | 1.26 | 0.026 |
| N2-O3 | π | O1-N2 | π^*^ | 6.16 | 1.07 | 0.073 |
| N2-O3 | π | N2-C4 | π^*^ | 3.66 | 0.96 | 0.055 |
| N2-O3 | π | C4-C14 | π^*^ | 0.66 | 1.28 | 0.026 |
| N2-C4 | π | O1-N2 | π^*^ | 5.99 | 0.97 | 0.068 |
| N2-C4 | π | N2-O3 | π^*^ | 5.96 | 0.97 | 0.068 |
| N2-C4 | π | N2-C4 | π^*^ | 0.73 | 0.85 | 0.023 |
| N2-C4 | π | C4-C5 | π^*^ | 1.85 | 1.15 | 0.041 |
| C4-C5 | π | C4-C14 | π^*^ | 0.55 | 1.16 | 0.023 |
| C4-C5 | π | C6-C7 | π^*^ | 0.89 | 1.14 | 0.028 |
| C4-C5 | π | C6-H23 | π^*^ | 1.94 | 1.05 | 0.040 |
| C4-C14 | π | N2-O3 | π^*^ | 0.70 | 0.96 | 0.023 |
| C4-C14 | π | N2-C4 | π^*^ | 2.62 | 0.85 | 0.044 |
| C4-C14 | π | C5-H22 | π^*^ | 1.55 | 1.06 | 0.036 |
| C4-C14 | π | C5-C6 | π^*^ | 19.59 | 0.29 | 0.068 |
| C5-C6 | π | N2-C4 | π^*^ | 2.60 | 0.84 | 0.043 |
| C5-C6 | π | C6-H23 | π^*^ | 0.57 | 1.05 | 0.022 |
| C5-C6 | π | C7-C8 | π^*^ | 1.72 | 1.01 | 0.038 |
| C5-C6 | π | C4-C14 | π^*^ | 22.19 | 0.28 | 0.070 |
| C5-H22 | π | N2-C4 | π^*^ | 1.89 | 0.72 | 0.034 |
| C5-H22 | π | C4-C5 | π^*^ | 0.92 | 1.03 | 0.028 |
| C5-H22 | π | C4-C14 | π^*^ | 1.98 | 1.05 | 0.041 |
| C5-H22 | π | C5-C6 | π^*^ | 0.77 | 1.05 | 0.025 |
| C5-H22 | π | C6-C7 | π^*^ | 2.11 | 1.03 | 0.042 |
| C5-H22 | π | C6-H23 | π^*^ | 0.76 | 0.94 | 0.024 |
| C6-C7 | π | C4-C5 | π^*^ | 0.88 | 1.13 | 0.028 |
| C6-C7 | π | C5-H22 | π^*^ | 1.98 | 1.04 | 0.041 |
| C6-C7 | π | C6-H23 | π^*^ | 0.66 | 1.04 | 0.023 |
| C6-C7 | π | C7-C8 | π^*^ | 0.77 | 1.00 | 0.025 |
| C6-H23 | π | C4-C5 | π^*^ | 2.18 | 1.03 | 0.042 |
| C7-C8 | π | C5-C6 | π^*^ | 1.68 | 1.14 | 0.039 |
| C7-C8 | π | C6-C7 | π^*^ | 1.14 | 1.12 | 0.032 |
| C7-C8 | π | C6-H23 | π^*^ | 0.63 | 1.02 | 0.023 |
| C7-C13 | π | C4-C14 | π^*^ | 0.74 | 1.16 | 0.026 |
| C7-C13 | π | C5-C6 | π^*^ | 0.71 | 1.16 | 0.026 |
| C7-C13 | π | C6-C7 | π^*^ | 0.59 | 1.14 | 0.023 |
| C7-C13 | π | C6-H23 | π^*^ | 1.65 | 1.05 | 0.037 |
| C7-C13 | π | C7-C8 | π^*^ | 1.30 | 1.01 | 0.033 |
| C7-C13 | π | C4-C14 | π^*^ | 18.78 | 0.28 | 0.066 |
| C7-C13 | π | C5-C6 | π^*^ | 20.46 | 0.28 | 0.070 |
| C8-O9 | π | C7-C8 | π^*^ | 1.20 | 1.19 | 0.034 |
| C8-O10 | π | C6-C7 | π^*^ | 1.06 | 1.16 | 0.031 |
| C8-O10 | π | C7-C8 | π^*^ | 2.69 | 1.02 | 0.047 |
| O10-C11 | π | C7-C8 | π^*^ | 1.15 | 1.07 | 0.032 |
| O1 | LP | 0.64 | π^*^ | 0.59 | 0.018 |  |
| N2 | LP | O1-O3 | π^*^ | 360.58 | 0.13 | 0.193 |

**Table S12.** Second-order pertbances theory analysis of NBO charges of ZAK-I-57.

| **Donor (i)** | **Type** | **Acceptor (j)** | **Type** | **E(2) Kcal/mol** | **E(j)-E(i) a.u** | **F(i.j) a.u** |
| --- | --- | --- | --- | --- | --- | --- |
| O1-C12 | π | C11-N12 | π^*^ | 3.84 | 1.18 | 0.060 |
| O1-N2 | π | N2-C4 | π^*^ | 3.66 | 0.96 | 0.055 |
| N2-C4 | π | C5-C6 | π^*^ | 1.36 | 1.18 | 0.036 |
| C5-C6 | π | C4-C14 | π^*^ | 0.69 | 1.16 | 0.025 |
| C5-C6 | π | C5-H22 | π^*^ | 0.57 | 1.05 | 0.022 |
| O10-C11 | π | C11-C15 | π^*^ | 2.67 | 1.09 | 0.048 |
| O10-C11 | π | C15-C21 | π^*^ | 0.90 | 1.22 | 0.030 |
| C11-N12 | π | C7-C13 | π^*^ | 1.15 | 1.20 | 0.033 |
| C11-N12 | π | O10-C11 | π^*^ | 3.77 | 0.99 | 0.056 |
| C11-N12 | π | C11-C15 | π^*^ | 1.40 | 1.10 | 0.035 |
| C11-N12 | π | N12-C13 | π^*^ | 0.98 | 1.08 | 0.029 |
| C11-N12 | π | C13-C14 | π^*^ | 1.89 | 1.20 | 0.043 |
| C11-N12 | π | C15-C16 | π^*^ | 0.80 | 1.23 | 0.028 |
| C11-N12 | π | C7-C13 | π^*^ | 15.73 | 0.31 | 0.068 |
| C11-N12 | π | C11-N12 | π^*^ | 1.41 | 0.30 | 0.019 |
| C11-N12 | π | C15-C16 | π^*^ | 8.21 | 0.34 | 0.050 |
| C11-C15 | π | C8-O10 | π^*^ | 1.70 | 0.81 | 0.034 |
| C11-C15 | π | O10-C11 | π^*^ | 2.29 | 0.87 | 0.041 |
| C11-C15 | π | C11-N12 | π^*^ | 0.72 | 1.07 | 0.025 |
| C11-C15 | π | N12-C13 | π^*^ | 2.26 | 0.97 | 0.042 |
| C11-C15 | π | C15-C16 | π^*^ | 0.98 | 1.12 | 0.030 |
| C11-C15 | π | C15-C21 | π^*^ | 1.00 | 1.12 | 0.030 |
| C11-C15 | π | C16-C17 | π^*^ | 1.57 | 1.13 | 0.038 |
| C11-C15 | π | C16-H25 | π^*^ | 0.65 | 1.02 | 0.023 |
| C11-C15 | π | C20-C21 | π^*^ | 1.58 | 1.13 | 0.038 |
| C11-C15 | π | C21-H28 | π^*^ | 0.66 | 1.02 | 0.023 |
| N12-C13 | π | C4-C14 | π^*^ | 1.42 | 1.14 | 0.036 |
| N12-C13 | π | C6-C7 | π^*^ | 1.41 | 1.12 | 0.036 |
| N12-C13 | π | C7-C8 | π^*^ | 0.86 | 0.99 | 0.026 |
| N12-C13 | π | C7-C13 | π^*^ | 1.38 | 1.10 | 0.035 |
| N12-C13 | π | O10-C11 | π^*^ | 3.74 | 0.90 | 0.052 |
| N12-C13 | π | C11-N12 | π^*^ | 1.75 | 1.09 | 0.039 |
| N12-C13 | π | C11-C15 | π^*^ | 3.41 | 1.01 | 0.052 |
| N12-C13 | π | C13-C14 | π^*^ | 1.22 | 1.11 | 0.033 |
| N12-C13 | π | C14-H24 | π^*^ | 0.60 | 1.03 | 0.022 |
| C13-C14 | π | N2-C4 | π^*^ | 2.85 | 0.83 | 0.045 |
| C13-C14 | π | C4-C5 | π^*^ | 0.76 | 1.13 | 0.026 |
| C13-C14 | π | C6-C7 | π^*^ | 0.71 | 1.13 | 0.025 |
| C13-C14 | π | C7-C8 | π^*^ | 1.78 | 1.00 | 0.038 |
| C13-C14 | π | C7-C13 | π^*^ | 0.57 | 1.11 | 0.023 |
| C13-C14 | π | C11-N12 | π^*^ | 1.15 | 1.11 | 0.032 |
| C13-C14 | π | N12-C13 | π^*^ | 1.03 | 1.00 | 0.029 |
| C13-C14 | π | C14-H24 | π^*^ | 0.65 | 1.04 | 0.023 |

**Table S13.** Second-order pertbances theory analysis of NBO charges of ZAK-I-64.

| **Donor (i)** | **Type** | **Acceptor (j)** | **Type** | **E(2) Kcal/mol** | **E(j)-E(i) a.u** | **F(i.j) a.u** |
| --- | --- | --- | --- | --- | --- | --- |
| N2-C4 | π | C4-C14 | π^*^ | 1.70 | 1.18 | 0.040 |
| N2-C4 | π | C5-H22 | π^*^ | 0.57 | 1.06 | 0.022 |
| C4-C5 | π | N2-C4 | π^*^ | 2.73 | 0.84 | 0.044 |
| C1-C2 | π | C11-C15 | π^*^ | 1.24 | 1.00 | 0.032 |
| C15-C16 | π | C16-H25 | π^*^ | 0.65 | 1.04 | 0.023 |
| C15-C16 | π | C17-C18 | π^*^ | 0.85 | 1.12 | 0.028 |
| C15-C16 | π | C17-H26 | π^*^ | 1.86 | 1.04 | 0.039 |
| C15-C16 | π | C20-C21 | π^*^ | 0.76 | 1.16 | 0.026 |
| C15-C16 | π | C21-H28 | π^*^ | 1.75 | 1.04 | 0.038 |
| C15-C16 | π | C11-N12 | π^*^ | 18.31 | 0.24 | 0.061 |
| C15-C16 | π | C17-C18 | π^*^ | 22.59 | 0.26 | 0.069 |
| C15-C16 | π | C20-C21 | π^*^ | 21.97 | 0.28 | 0.071 |
| C15-C21 | π | O10-C11 | π^*^ | 1.56 | 0.89 | 0.034 |
| C15-C21 | π | C11-N12 | π^*^ | 0.70 | 1.09 | 0.025 |
| C15-C21 | π | C11-C15 | π^*^ | 1.01 | 1.00 | 0.029 |
| C15-C21 | π | C16-C17 | π^*^ | 0.78 | 1.15 | 0.027 |
| C15-C21 | π | C16-H25 | π^*^ | 1.83 | 1.04 | 0.039 |
| C15-C21 | π | C18-C20 | π^*^ | 0.86 | 1.12 | 0.028 |
| C15-C21 | π | C20-H27 | π^*^ | 1.88 | 1.03 | 0.039 |
| C15-C21 | π | C21-H28 | π^*^ | 0.60 | 1.04 | 0.022 |
| C16-C17 | π | C11-C15 | π^*^ | 1.90 | 1.01 | 0.039 |
| C16-C17 | π | C15-C21 | π^*^ | 0.74 | 1.14 | 0.026 |
| C16-C17 | π | C16-H25 | π^*^ | 0.60 | 1.04 | 0.022 |
| C16-C17 | π | C17-H26 | π^*^ | 0.57 | 1.04 | 0.022 |
| C16-C17 | π | C18-Br19 | π^*^ | 2.21 | 0.76 | 0.037 |
| C16-C17 | π | C18-C20 | π^*^ | 0.73 | 1.13 | 0.026 |
| C16-H25 | π | C11-C15 | π^*^ | 1.16 | 0.90 | 0.029 |
| C16-H25 | π | C15-C16 | π^*^ | 0.92 | 1.04 | 0.028 |
| C16-H25 | π | C15-C21 | π^*^ | 1.95 | 1.04 | 0.040 |
| C16-H25 | π | C16-C17 | π^*^ | 0.74 | 1.05 | 0.025 |
| C16-H25 | π | C17-C18 | π^*^ | 2.28 | 1.02 | 0.043 |
| C16-H25 | π | C17-H26 | π^*^ | 0.80 | 0.94 | 0.024 |
| C17-C18 | π | C15-C16 | π^*^ | 0.81 | 1.15 | 0.027 |
| C17-C18 | π | C16-H25 | π^*^ | 1.78 | 1.05 | 0.039 |
| C17-C18 | π | C17-H26 | π^*^ | 0.64 | 1.05 | 0.023 |
| C17-C18 | π | C18-Br19 | π^*^ | 2.98 | 0.76 | 0.043 |
| C17-C18 | π | C20-C21 | π^*^ | 0.78 | 1.17 | 0.027 |
| C17-C18 | π | C20-H27 | π^*^ | 1.80 | 1.05 | 0.039 |
| C17-C18 | π | C15-C16 | π^*^ | 22.58 | 0.29 | 0.072 |
| C17-C18 | π | C20-C21 | π^*^ | 19.37 | 0.29 | 0.068 |
| C17-H26 | π | C15-C16 | π^*^ | 2.19 | 1.04 | 0.043 |
| C17-H26 | π | C16-C17 | π^*^ | 0.65 | 1.05 | 0.023 |
| C17-H26 | π | C16-H25 | π^*^ | 0.78 | 0.94 | 0.024 |
| C17-H26 | π | C17-C18 | π^*^ | 1.01 | 1.03 | 0.029 |
| C17-H26 | π | C18-Br19 | π^*^ | 1.08 | 0.65 | 0.024 |
| C17-H26 | π | C18-C20 | π^*^ | 1.84 | 1.02 | 0.039 |
| C18-Br19 | π | C16-C17 | π^*^ | 2.04 | 1.06 | 0.042 |
| O1 | LP | N2-O3 | π^*^ | 15.92 | 0.49 | 0.080 |
| N2 | LP | C4-C14 | π^*^ | 13.99 | 0.30 | 0.064 |

**Table S14.** Second-order pertbances theory analysis of NBO charges of ZAK-I-68.

| **Donor (i)** | **Type** | **Acceptor (j)** | **Type** | **E(2) Kcal/mol** | **E(j)-E(i) a.u** | **F(i.j) a.u** |
| --- | --- | --- | --- | --- | --- | --- |
| N2-C4 | π | C4-C14 | π^*^ | 1.70 | 1.12 | 0.042 |
| N2-C4 | π | C5-H22 | π^*^ | 0.53 | 1.06 | 0.022 |
| C4-C5 | π | N2-C4 | π^*^ | 2.73 | 0.84 | 0.044 |
| C1-C2 | π | C15-C21 | π^*^ | 0.82 | 1.15 | 0.027 |
| C18-C2 | π | C16-C17 | π^*^ | 0.79 | 1.16 | 0.027 |
| C18-C20 | π | C17-H26 | π^*^ | 1.81 | 1.04 | 0.039 |
| C18-C20 | π | C20-H27 | π^*^ | 0.64 | 1.05 | 0.023 |
| C18-C20 | π | C21-H28 | π^*^ | 1.79 | 1.05 | 0.039 |
| C20-C21 | π | C11-C15 | π^*^ | 1.83 | 1.01 | 0.039 |
| C20-C21 | π | C15-C16 | π^*^ | 0.72 | 1.14 | 0.026 |
| C20-C21 | π | C17-C18 | π^*^ | 0.73 | 1.13 | 0.026 |
| C20-C21 | π | C18-Br19 | π^*^ | 2.21 | 0.76 | 0.037 |
| C20-C21 | π | C20-H27 | π^*^ | 0.57 | 1.04 | 0.022 |
| C20-C21 | π | C21-H28 | π^*^ | 0.62 | 1.05 | 0.023 |
| C20-C21 | π | C15-C16 | π^*^ | 19.21 | 0.28 | 0.066 |
| C20-C21 | π | C17-C18 | π^*^ | 21.99 | 0.27 | 0.069 |
| C20-H27 | π | C15-C21 | π^*^ | 2.20 | 1.04 | 0.043 |
| C20-H27 | π | C17-C18 | π^*^ | 1.84 | 1.02 | 0.039 |
| C20-H27 | π | C18-Br19 | π^*^ | 1.08 | 0.65 | 0.024 |
| C20-H27 | π | C18-C20 | π^*^ | 1.03 | 1.02 | 0.029 |
| C20-H27 | π | C20-C21 | π^*^ | 0.64 | 1.06 | 0.023 |
| C20-H27 | π | C21-H28 | π^*^ | 0.78 | 0.95 | 0.024 |
| C21-H28 | π | C11-C15 | π^*^ | 1.18 | 0.90 | 0.029 |
| C21-H28 | π | C15-C16 | π^*^ | 2.00 | 1.03 | 0.041 |
| C21-H28 | π | C15-C21 | π^*^ | 0.86 | 1.03 | 0.027 |
| C21-H28 | π | C18-C20 | π^*^ | 2.31 | 1.02 | 0.043 |
| C21-H28 | π | C20-C21 | π^*^ | 0.79 | 1.05 | 0.026 |
| C21-H28 | π | C20-H27 | π^*^ | 0.81 | 0.93 | 0.025 |
| O3 | LP | O1-N2 | π^*^ | 15.86 | 0.49 | 0.080 |
| O3 | LP | N2-C4 | π^*^ | 15.01 | 0.38 | 0.068 |
| O1 | LP | N2-C4 | π^*^ | 15.09 | 0.38 | 0.068 |
| N2 | LP | C4-C14 | π^*^ | 13.99 | 0.30 | 0.064 |

**Table S15.** Second-order pertbances theory analysis of NBO charges of ZAK-I-87.

| **Donor (i)** | **Type** | **Acceptor (j)** | **Type** | **E(2) Kcal/mol** | **E(j)-E(i) a.u** | **F(i.j) a.u** |
| --- | --- | --- | --- | --- | --- | --- |
| N2-C4 | π | N2-O3 | π^*^ | 1.88 | 0.11 | 0.039 |
| N2-C4 | π | C5-C6 | π^*^ | 1.04 | 0.33 | 0.056 |
| N2-C4 | π | C5-H22 | π^*^ | 1.35 | 0.21 | 0.051 |
| N2-C4 | π | C13-C14 | π^*^ | 1.37 | 0.29 | 0.059 |
| N2-C4 | π | C14-H24 | π^*^ | 1.44 | 0.22 | 0.053 |
| C7-C13 | π | C5-C6 | π^*^ | 187.97 | 0.01 | 0.075 |
| C8-O10 | π | C6-C7 | π^*^ | 0.98 | 0.28 | 0.048 |
| C8-O10 | π | C7-C8 | π^*^ | 0.65 | 0.15 | 0.024 |
| C8-O10 | π | C7-C13 | π^*^ | 1.19 | 0.26 | 0.049 |
| C8-O10 | π | C8-O9 | π^*^ | 0.68 | 0.30 | 0.040 |
| C8-O10 | π | O10-C11 | π^*^ | 11.31 | 0.06 | 0.065 |
| C8-O10 | π | C11-N12 | π^*^ | 0.69 | 0.26 | 0.037 |
| C8-O10 | π | C11-C15 | π^*^ | 0.89 | 0.17 | 0.033 |
| C11-N12 | π | C15-C16 | π^*^ | 47.89 | 0.03 | 0.061 |
| C17-C18 | π | C15-C16 | π^*^ | 261.36 | 0.01 | 0.079 |
| C17-C18 | π | C20-C21 | π^*^ | 140.35 | 0.02 | 0.074 |
| O3 | LP | C5-H22 | π^*^ | 0.78 | 0.59 | 0.020 |
| O9 | LP | C7-C8 | π^*^ | 21.64 | 0.56 | 0.101 |
|  | LP |  | π^*^ |  |  |  |

**Table S16.** Physicochemical properties of benzoxazinone derivatives.

| **Compound IDs** | **MW** | **nRot** | **HBA** | **HBD** | **MR** | **TPSA** | **M log P** | **Ali log S** |
| --- | --- | --- | --- | --- | --- | --- | --- | --- |
| ZAK-I-55 | 347.12 | 2 | 5 | 0 | 82.24 | 88.92 | 3.34 | -5.00 |
| ZAK-I-57 | 318.28 | 2 | 5 | 0 | 92.04 | 88.92 | 3.50 | -5.58 |
| ZAK-I-64 | 282.25 | 2 | 5 | 0 | 79.5 | 88.92 | 2.14 | -4.66 |
| ZAK-I-68 | 284.22 | 2 | 6 | 1 | 76.56 | 109.15 | 1.08 | -4.33 |
| ZAK-I-87 | 286.21 | 2 | 6 | 0 | 74.5 | 88.92 | 3.09 | -4.39 |
| ZAK-I-90 | 302.67 | 2 | 5 | 0 | 79.55 | 88.92 | 3.22 | -4.93 |
| ZAK-I-93 | 312.25 | 3 | 6 | 0 | 84.43 | 88.92 | 3.25 | -4.82 |
| ZAK-I-97 | 354.31 | 5 | 7 | 0 | 97.46 | 107.38 | 1.48 | -5.04 |

**Optimal range:** MW ≤ 600, nRot ≤ 10, HBA ≤ 10, HBD ≤ 5, MR ≤ 155, TPSA ≤ 150 Å^2^, M log P ≤ 5, Ali log S ≤ 0.^16,17^

**Table S17.** Drug-likeness and medicinal chemistry properties of benzoxazinone derivatives.

| **Compound ID** | **Lipinski** | **Bioavailability Score** | **PAINS** | **Brenk** | **Leadlikeness** | **Synthetic accessibility** |
| --- | --- | --- | --- | --- | --- | --- |
| ZAK-I-55 | Yes | 0.55 | 0 | 2 | Yes | 2.96 |
| ZAK-I-57 | Yes | 0.55 | 0 | 2 | No (1 violation) | 3.12 |
| ZAK-I-64 | Yes | 0.55 | 0 | 2 | Yes | 3.09 |
| ZAK-I-68 | Yes | 0.55 | 0 | 2 | Yes | 2.86 |
| ZAK-I-87 | Yes | 0.55 | 0 | 2 | Yes | 2.92 |
| ZAK-I-90 | Yes | 0.55 | 0 | 2 | Yes | 2.89 |
| ZAK-I-93 | Yes | 0.55 | 0 | 2 | Yes | 3.11 |
| ZAK-I-97 | Yes | 0.55 | 0 | 2 | No; (1 violation) | 3.31 |

**Table S18.** Pharmacokinetic properties of the benzoxazinone derivatives.

| **Compound ID** | **GI absorption** | **BBB permeant** | **P-gp substrate** | **CYP1A2 inhibitor** | **CYP2C19 inhibitor** | **CYP2C9 inhibitor** | **CYP2D6 inhibitor** | **CYP3A4 inhibitor** | **Log *K*_p_ (skin permeation)** |
| --- | --- | --- | --- | --- | --- | --- | --- | --- | --- |
| ZAK-I-55 | High | No | No | Yes | Yes | Yes | No | No | -5.97 |
| ZAK-I-57 | High | No | No | Yes | Yes | Yes | No | No | -5.39 |
| ZAK-I-64 | High | No | No | Yes | No | Yes | No | No | -5.81 |
| ZAK-I-68 | High | No | No | Yes | No | No | No | No | -6.33 |
| ZAK-I-87 | High | No | No | Yes | Yes | No | No | No | -6.02 |
| ZAK-I-90 | High | No | No | Yes | Yes | Yes | No | No | -5.75 |
| ZAK-I-93 | High | No | No | Yes | Yes | Yes | No | No | -5.88 |
| ZAK-I-97 | High | No | No | Yes | Yes | Yes | No | No | -6.25 |

**Table S19.** Intersecting targets between HCC-related and potential protein targets of benzoxazinone derivatives.

| **Serial No.** | **Intersecting targets** |
| --- | --- |
| 1 | ABCG2 |
| 2 | ADAM17 |
| 3 | AR |
| 4 | AURKA |
| 5 | C1R |
| 6 | CA1 |
| 7 | CA2 |
| 8 | CASP1 |
| 9 | CASP3 |
| 10 | CCNA2 |
| 11 | CCND1 |
| 12 | CCNE1 |
| 13 | CDC25A |
| 14 | CDC25B |
| 15 | CDK4 |
| 16 | CES1 |
| 17 | CES2 |
| 18 | COMT |
| 19 | CYP2C9 |
| 20 | CYP3A4 |
| 21 | DDX3X |
| 22 | EGFR |
| 23 | ERBB2 |
| 24 | ESR1 |
| 25 | GSK3A |
| 26 | GSTP1 |
| 27 | HSP90AA1 |
| 28 | MAP2K1 |
| 29 | MAPK1 |
| 30 | MET |
| 31 | MIF |
| 32 | MME |
| 33 | MMP2 |
| 34 | MMP3 |
| 35 | MMP9 |
| 36 | MYC |
| 37 | PARP1 |
| 38 | PGK1 |
| 39 | PIK3CA |
| 40 | PLAU |
| 41 | PLG |
| 42 | PLK1 |
| 43 | PTGS2 |
| 44 | PTK2 |
| 45 | RAF1 |
| 46 | RET |
| 47 | SGK1 |
| 48 | SRC |
| 49 | TERT |
| 50 | TTR |

**Table S20.** The molecular docking results were expressed in the form of binding energies.

| **Compound ID** |  | **Binding affinity (kcal/mol)** | | | |  |
| --- | --- | --- | --- | --- | --- | --- |
|  | **c-Myc** | **ESR1** | **EGFR** | **HSP90AA1** | **CCND1** | **ERBB2** |
| ZAK-I-57 | -6.6 | -9.4 | -8.0 | -9.3 | -8.0 | -6.3 |
| ZAK-I-64 | -6.3 | -9.0 | -7.1 | -10.1 | -7.6 | -5.8 |
| ZAK-I-68 | -6.5 | -8.9 | -7.4 | -9.5 | -7.5 | -5.9 |
| ZAK-I-87 | -6.0 | -8.5 | -7.0 | -9.8 | -7.6 | -5.4 |
| ZAK-I-93 | -5.7 | -8.0 | -6.9 | -8.2 | -7.2 | -5.8 |

**Table S21.** Comparative analysis of different physiochemical properties between ZAK-I-57 and Sorafenib.

| Property | Sorafenib | ZAK-I-57 | Implication for Drug Performance |
| --- | --- | --- | --- |
| Molecular Formula | C_21_H_16_ClF_3_N_4_O_3_ | C_18_H_10_N_2_O_4_ | Different scaffolds: ZAK-I-57 has a simpler, more aromatic structure. |
| Molecular Weight (MW) | 464.82 g/mol | 318.28 g/mol | ZAK-I-57’s lower MW favors better oral bioavailability and permeability (Lipinski’s Rule of Five). |
| Heavy Atoms | 32 | 24 | Fewer heavy atoms in ZAK-I-57 suggest improved pharmacokinetics and reduced metabolic complexity. |
| Aromatic Heavy Atoms | 18 | 20 | ZAK-I-57’s higher aromaticity supports stronger π–π stacking interactions with oncogenic targets. |
| Fraction Csp3 | 0.10 | 0.00 | ZAK-I-57’s full aromaticity enhances planar interactions with target proteins. |
| Rotatable Bonds | 9 | 2 | ZAK-I-57’s rigidity reduces entropy loss upon binding, improving binding affinity. |
| H-Bond Acceptors | 7 | 5 | Fewer acceptors in ZAK-I-57 suggest a more compact and efficient hydrogen bonding profile. |
| H-Bond Donors | 3 | 0 | ZAK-I-57 lacks H-bond donors, favoring membrane permeability over solubility. |
| Molar Refractivity | 112.48 | 92.04 | Lower refractivity in ZAK-I-57 suggests favorable ligand-target interactions. |
| TPSA | 92.35 Å² | 88.92 Å² | Both values are within the optimal range (≤140 Å²) for cell membrane permeability and bioavailability. |

**References**

1. Khan SA, Rizwan K, Shahid S, Noamaan MA, Rasheed T, Amjad H. Synthesis, DFT, computational exploration of chemical reactivity, molecular docking studies of novel formazan metal complexes and their biological applications. *Appl Organomet Chem*. 2020;34(3):e5444. Accessed September 6, 2021. https://onlinelibrary.wiley.com/doi/full/10.1002/aoc.5444

2. Daina A, Michielin O, Zoete V. SwissADME: a free web tool to evaluate pharmacokinetics, drug-likeness and medicinal chemistry friendliness of small molecules. *Scientific Reports 2017 7:1*. 2017;7(1):1-13. doi:10.1038/srep42717

3. Daina A, Michielin O, Zoete V. SwissTargetPrediction: updated data and new features for efficient prediction of protein targets of small molecules. *Nucleic Acids Res*. 2019;47(W1):W357-W364. doi:10.1093/NAR/GKZ382

4. Su WH, Chao CC, Yeh SH, Chen DS, Chen PJ, Jou YS. OncoDB.HCC: an integrated oncogenomic database of hepatocellular carcinoma revealed aberrant cancer target genes and loci. *Nucleic Acids Res*. 2007;35(suppl_1):D727-D731. doi:10.1093/NAR/GKL845

5. Lee L, Wang K, Li G, et al. Liverome: A curated database of liver cancer-related gene signatures with self-contained context information. *10th Int Conference on Bioinformatics - 1st ISCB Asia Joint Conference 2011, InCoB 2011/ISCB-Asia 2011: Computational Biology - Proceedings from Asia Pacific Bioinformatics Network (APBioNet)*. 2011;12(SUPPL. 3):1-13. doi:10.1186/1471-2164-12-S3-S3/FIGURES/5

6. Wang Q, Liang Y, Peng C, Jiang P. Network Pharmacology-Based Study on the Mechanism of Scutellariae Radix for Hepatocellular Carcinoma Treatment. *Evid Based Complement Alternat Med*. 2020;2020. doi:10.1155/2020/8897918

7. Oliveros JC. Venny 2.1.0. 2007. Accessed March 17, 2022. https://bioinfogp.cnb.csic.es/tools/venny/

8. von Mering C, Huynen M, Jaeggi D, Schmidt S, Bork P, Snel B. STRING: a database of predicted functional associations between proteins. *Nucleic Acids Res*. 2003;31(1):258-261. doi:10.1093/NAR/GKG034

9. Lopes CT, Franz M, Kazi F, et al. Cytoscape Web: an interactive web-based network browser. *Bioinformatics*. 2010;26(18):2347-2348. doi:10.1093/BIOINFORMATICS/BTQ430

10. GEPIA (Gene Expression Profiling Interactive Analysis). Accessed April 28, 2022. http://gepia.cancer-pku.cn/

11. DAVID Functional Annotation Bioinformatics Microarray Analysis. Accessed April 19, 2022. https://david.ncifcrf.gov/

12. Weishengxin. Weishengxin - data analysis and visualization experts around you. Accessed March 17, 2022. http://www.bioinformatics.com.cn/

13. RCSB PDB: Search. Accessed February 11, 2022. https://www.rcsb.org/search

14. BIOVIA DS. BIOVIA discovery studio visualizer. *2016*. 20:779.

15. Trott O, Olson AJ. AutoDock Vina: Improving the speed and accuracy of docking with a new scoring function, efficient optimization, and multithreading. *J Comput Chem*. 2010;31(2):455-461. doi:10.1002/JCC.21334

16. Zafar F, Gupta A, Thangavel K, et al. Physicochemical and Pharmacokinetic Analysis of Anacardic Acid Derivatives. *ACS Omega*. 2020;5(11):6021-6030. doi:10.1021/ACSOMEGA.9B04398/ASSET/IMAGES/LARGE/AO9B04398_0004.JPEG

17. Veber DF, Johnson SR, Cheng HY, Smith BR, Ward KW, Kopple KD. Molecular properties that influence the oral bioavailability of drug candidates. *J Med Chem*. 2002;45(12):2615-2623. doi:10.1021/JM020017N/SUPPL_FILE/JM020017N_S.PDF
